# Detection and Capture of Volatile Fluorinated Compounds Using Porous Materials

**DOI:** 10.3390/nano16171125

**Published:** 2026-09-07

**Authors:** Jiejing Hou, Xinlei Tao, Ce Zhang, Zidan Zhang, Huihui Kong, Qingmin Ji, Hengdao Quan, Harald Fuchs

**Affiliations:** 1 Herbert Gleiter Institute of Nanoscience, School of Materials Science and Engineering, Nanjing University of Science and Technology, 200 Xiaolingwei, Nanjing 210094, China; houjiejing@njust.edu.cn (J.H.); zhangce@njust.edu.cn (C.Z.); 125116023965@njust.edu.cn (Z.Z.); konghuihui@njust.edu.cn (H.K.); fuchsh@uni-muenster.de (H.F.); 2 School of Life Sciences and Technology, China Pharmaceutical University, 639 Longmian Avenue, Nanjing 211198, China; leotaox@163.com; 3 School of Materials and Chemistry, University of Shanghai for Science and Technology, 516 Jungong Road, Shanghai 200093, China; 4 Center for Nanotechnology, University of Münster, Heisenbergstrasse 11, Münster 48149, Germany

**Keywords:** volatile fluorinated compounds, fluorinated gas sensing, fluorinated gas capture, functional porous materials, selective adsorption

## Abstract

Volatile fluorinated compounds (VFCs) are indispensable to modern industry, yet their potent greenhouse effects pose critical environmental challenges. Functional porous materials, ranging from zeolites and semiconductor oxides to metal–organic frameworks (MOFs), covalent organic frameworks (COFs), and other advanced porous materials, have emerged as versatile platforms for VFC sensing and capture, leveraging their structural tunability, ultrahigh surface areas, and designable pore chemistry. This review provides a systematic summary of recent advances in porous materials for VFC management. For sensing, we examine transduction mechanisms (chemiresistive, optical, and gravimetric approaches) with emphasis on structure–signal relationships. For capture, we evaluate adsorptive performance across VFC subclasses, highlighting design principles that govern selectivity and capacity. Based on recent achievements, we assess persistent gaps between laboratory-scale achievements and practical deployment. Possible pathways toward integrated sense-and-capture systems are also explored. By bridging fundamental materials science, this review highlights cross-cutting design strategies that may accelerate the development of next-generation VFC management platforms.

## 1. Introduction

Volatile fluorinated compounds (VFCs) serve as critical industrial media for modern high-tech manufacturing. This family includes permanent gases like sulfur hexafluoride (SF_6_) and nitrogen trifluoride (NF_3_), along with vapor-phase chlorofluorocarbons (CFCs), hydrofluorocarbons (HFCs), perfluorocarbons (PFCs), and fluorinated solvents. Their exceptional chemical inertness (particularly for fully fluorinated species, such as SF_6_, NF_3_, and PFCs), along with high dielectric strength and thermal stability, render them indispensable in plasma etching/chamber cleaning, high-voltage electrical insulation, and advanced refrigeration systems [1,2]. However, these unique physicochemical properties create an environmental paradox. Many commonly used VFCs persist in the atmosphere for decades to millennia, making them potent greenhouse gases [3]. The international community has enacted stringent regulatory frameworks, from the Kyoto Protocol to the Kigali Amendment and recent EU F-gas regulations, to mandate tighter controls on VFC emissions. These regulatory agreements have, in turn, stimulated extensive research into environmental impact assessment, low-global warming potential (GWP) alternatives, and advanced technologies for VFC sensing and capture [4]. Despite significant advances in alternatives, a complete replacement of conventional VFCs remains a long-term endeavor. Therefore, exploring robust monitoring and capture technologies is essential, not only for mitigating current emissions of VFCs but also for supporting the adoption of next-generation refrigerants.

Functional porous materials, spanning from traditional zeolites and semiconducting oxides to metal–organic frameworks (MOFs), covalent organic frameworks (COFs), hydrogen-bonded organic frameworks (HOFs), and porous organic cages (POCs), have emerged as the most promising candidates for gas sensing and capture [5,6,7,8,9]. Unlike conventional bulk materials, they offer structural tunability, high surface areas, and designable pore environments to target the specific features of VFCs. For example, strategies such as anion-pillaring, the incorporation of open metal sites, and fluorophilic functionalization can drastically enhance affinity and selectivity towards fluorinated species, even in the presence of humidity or competing gases [10,11]. The intrinsic electronic conjugation of certain conductive frameworks or their ability to host luminescent guests enables direct signal transduction (chemiresistive, optical, or gravimetric) upon gas adsorption [12]. Although sensing and capture are often studied separately, both processes hinge on a similar fundamental mechanism of host–guest interactions in nanopores.

While several reviews have summarized progress in VFC capture or sensing within specific material families [10,13,14,15], a cross-material comparison of different porous platforms across the full spectrum of major VFC gas families is still lacking. Such a comparative perspective is important for advancing the practical application of VFC adsorbents, where multiple factors, including synthesis scalability, adsorption performance, material cost, and operating conditions, must be considered simultaneously. Therefore, the extraction of cross-cutting structure–property relationships and the identification of persistent design challenges are essential but often overlooked in material-centric reviews. Furthermore, since sensing and capture may share structural design principles, a unified discussion should be beneficial for the growing demand for multifunctional devices.

**Figure 1:**
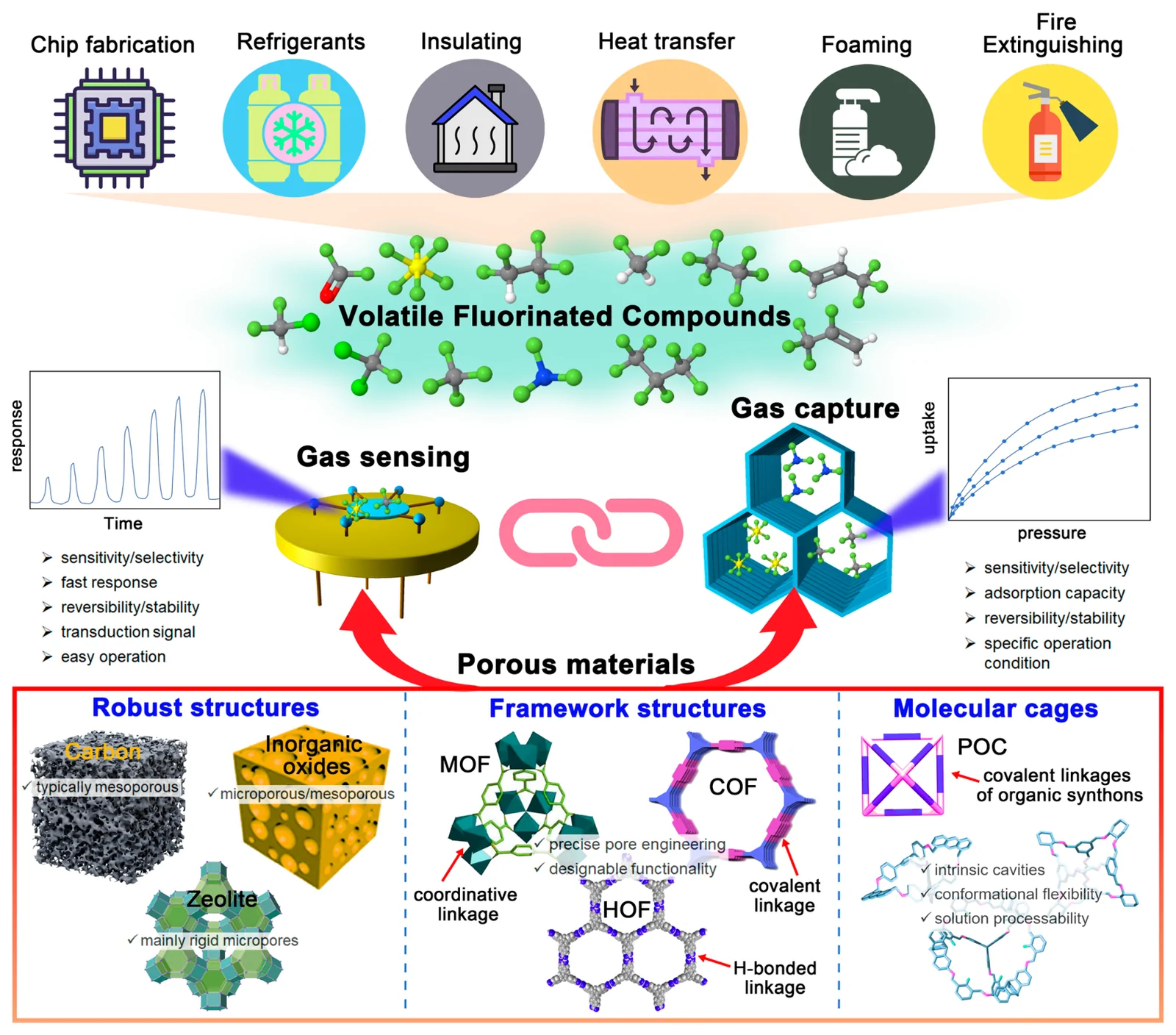
A scheme for porous materials applied for sensing and capturing volatile fluorinated compounds (VFCs) from industry applications. The green shaded areas display the molecular structures of representative VFCs. The red solid box at the bottom encloses different categories of porous structures, while the blue vertical dashed lines inside further subdivide them into three types. The small red arrows in the red box point to the predominant intermolecular forces for the assembly of each structural type. The thick red upward-pointing arrows indicate the dual functions of sensing and capture for VFCs. The two signal plots show the response signals for sensing and capture, using a blue gradient mark. A double-ring clasp in the center symbolizes the combined/synergistic nature of sensing and capture functionalities.

Guided by these considerations, this review summarizes recent progress in the development of diverse porous materials for the selective sensing and capture of VFCs. Figure 1 provides a schematic overview of the major VFC classes covered in this review, along with the functional requirements for sensing and capture and the porous material platforms discussed. We first consolidate the foundational design principles governing VFC–framework interactions, then survey the state-of-the-art in sensing and capture applications. For sensing, we examine different transduction mechanisms, including chemiresistive, optical, and gravimetric approaches. For capture, we evaluate adsorptive performance across the major VFC subclasses (e.g., SF_6_/NF_3_, PFCs, HFCs, and CFCs/HFOs). Recognizing the complementary nature of these functionalities, we further outline the prospect toward integrated “sense-and-capture” architectures. By bridging fundamental materials science with practical environmental engineering, this review aims to highlight cross-cutting design strategies that may accelerate the development of next-generation VFC management platforms.

## 2. Design Strategies of Functional Porous Structures for Capturing VFCs

The rational design of porous materials for gas management hinges on a deep understanding of the target molecules. Compared with the more extensively studied areas of CO_2_ capture and VOC sensing [16,17,18], VFCs present a distinct set of physicochemical characteristics. Unlike CO_2_, which possesses a significant quadrupole moment, or VOCs with high polarizability and reactivity, most VFCs are characterized by exceptional chemical inertness. Three electronic properties are particularly relevant to their recognition in porous frameworks. The large electronegativity difference between carbon and fluorine gives rise to significant localized negative charge on the fluorine atoms. Molecular symmetry then determines whether these local dipoles yield a net molecular dipole. In highly symmetric molecules, such as CF_4_, the individual C–F bond dipoles cancel vectorially, resulting in a zero net dipole. Meanwhile, for asymmetric VFCs, such as HFCs and HFOs, both local charge and a finite net dipole coexist, providing additional interaction pathways. The local charges on fluorine persist even in the absence of a net molecular dipole, enabling electrostatic recognition via interactions with Lewis acidic sites or cations. Independent of dipole moment, molecular polarizability varies considerably across the VFC family. Small perfluorocarbons have low polarizability, whereas larger species and those containing heavier atoms (e.g., SF_6_, NF_3_) exhibit substantially higher polarizability that can be exploited for selective adsorption.

The core mechanism underlying both sensing and capture of VFCs remains rooted in specific host–guest interactions, much like those exploited in other gas systems. Whether aiming for a measurable signal or for high-capacity capture, the initial step is always selective recognition and binding of the VFC molecule within the porous framework. This common foundation renders the determinants of adsorption affinity and selectivity inherently coupled, irrespective of the specific functional application.

**Figure 2:**
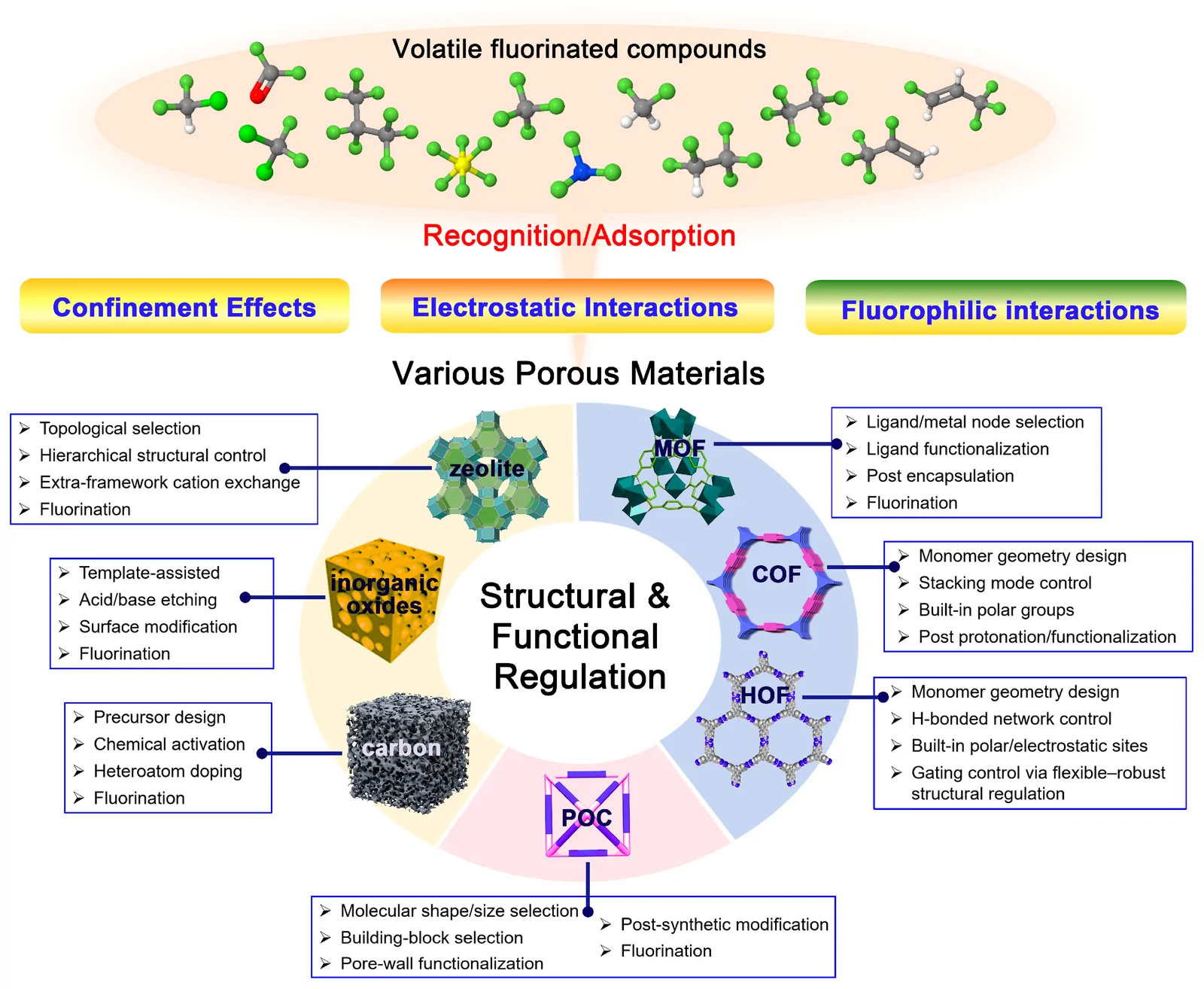
A summary of structural and functional regulation strategies of functional porous materials for capturing VFCs. The light pink shaded area at the top displays the molecular structures of various VFCs. The three yellow boxes below list different recognition/interaction types. The circular panels contain representative schematic illustrations of different porous material structures. The blue boxes summarize the corresponding functionalization strategies.

The recognition of VFCs by porous materials is governed by three primary interaction mechanisms (confinement effects, electrostatic interactions, and fluorophilic surface chemistry), as illustrated in Figure 2. These mechanisms are not mutually exclusive and often operate synergistically within a single material platform.

(1) Confinement effects. In the absence of strong electrostatic or chemical bonding, nanoscale confinement, where pore dimensions closely match the molecular size of VFCs, can substantially enhance adsorption through cumulative van der Waals interactions [19]. This “pore-filling” effect often yields high adsorption enthalpies, despite the molecule’s inherent inertness, and may be particularly effective for larger VFCs [20].
(2) Electrostatic interactions. Although many VFCs possess weak or zero permanent dipoles, they often exhibit significant quadrupole moments or highly localized negative charge densities on fluorine atoms. Materials engineered with strong local electric fields, such as those featuring open metal sites, extra-framework cations, or polar functional groups, can leverage ion–quadrupole or polarization-induced interactions, and dipole-induced dipole interactions to achieve selective binding [21].
(3) Fluorophilic interactions. Certain chemical micro-environments, exemplified by perfluorinated interfaces or particular metal coordination sites, exhibit a marked thermodynamic preference for fluorinated substrates relative to their non-fluorinated analogs [22,23,24]. This selectivity arises from the unique electronic characteristics and low surface energy of F, enabling effective recognition even in complex gas mixtures.

Achieving optimal VFC recognition requires precise control over two interdependent structural parameters of pore architecture and surface chemistry. Different classes of porous materials, from traditional zeolites and carbons to MOFs, COFs, HOFs, and POCs, may offer distinct capabilities and limitations in realizing these design strategies. Here, we compare the capabilities of different material platforms in achieving confinement, electrostatic interaction, and fluorophilic recognition. Figure 2 briefly provides an overview of how these three interaction mechanisms can be realized through different functionalization strategies across various porous material platforms.

### 2.1. Modulation Strategies for Pore Architecture

Pore architecture dictates molecular access and occupancy. The most direct strategy of “size matching” employs pore apertures slightly larger than the kinetic diameter of the target gases [25,26], maximizing confinement efficiency while excluding larger interferents. Reticular frameworks are well suited for this strategy. Their modular assembly through coordination bonds, covalent linkages, or hydrogen bonds allows for systematic regulation of pore dimensions with sub-angstrom precision, thereby enabling an ideal molecular-sieving effect [27]. These frameworks can be further classified by their dimensionality (1D, 2D, or 3D) based on the connectivity of their building units [28]. This structural hierarchy offers additional handles for tuning pore architecture and molecular transport pathways.

COFs distinguish themselves through fully covalent frameworks that combine high stability with tunable mesoporosity and multi-dimensional topologies [29,30]. Their structural versatility also enables integration of π-conjugated systems, positioning them as promising platforms for chemiresistive VFC sensing. However, achieving long-range crystallinity and uniform pore distribution remains a synthetic challenge.

HOFs represent a class of crystalline porous materials assembled via reversible hydrogen bonding [31]. Their high crystallinity enables precise structural elucidation of guest binding sites through single-crystal X-ray diffraction [32]. The dynamic nature of hydrogen bonds facilitates regeneration under mild conditions. Moreover, the solution processability of HOFs allows the fabrication into films or membranes, offering practical advantages over many insoluble frameworks. Nevertheless, the inherent fragility of networks can limit their stability under harsh conditions, and the pore sizes are also often restricted to the microporous regime.

POCs are discrete molecular entities with intrinsic cavities as extended frameworks. Their defined molecular structures and excellent solubility enable facile functionalization and solution processability [33]. Fluorine-functionalized POCs exhibit exceptional selectivity toward perfluorinated gases over non-fluorinated analogs, highlighting the importance of peripheral group engineering [34]. The molecular nature of POCs facilitates direct structure–property correlation studies [35,36], which is unavailable in extended networks. However, the reliance on weak intermolecular packing for porosity imposes inherent constraints on stability and pore tunability compared to MOFs or COFs.

Zeolites provide well-defined micropores and exceptional thermal stability. However, their topological diversity remains restricted, as most known frameworks feature aperture diameters below 7 Å [37]. This limitation makes them unsuitable for larger VFCs, such as PFCs (kinetic diameters typically > 6 Å).

Activated carbons are among the most widely used adsorbents in industrial gas separation, owing to their high surface area, low cost, and intrinsic hydrophobicity [38,39]. However, their broad pore size distribution and high surface energy may compromise selective adsorption for discriminating between different VFC species or against background interferents.

In contrast to these crystalline and discrete materials, amorphous POPs offer exceptional design flexibility and robust physicochemical stability [40,41]. While lacking the precise pore ordering of COFs, POPs compensate with high surface areas and diverse building blocks, allowing for tailored functionalization to enhance VFC affinity. However, their intrinsic structural disorder and broad pore size distributions often limit molecular-level discrimination and precise structure–property correlations.

**Figure 3:**
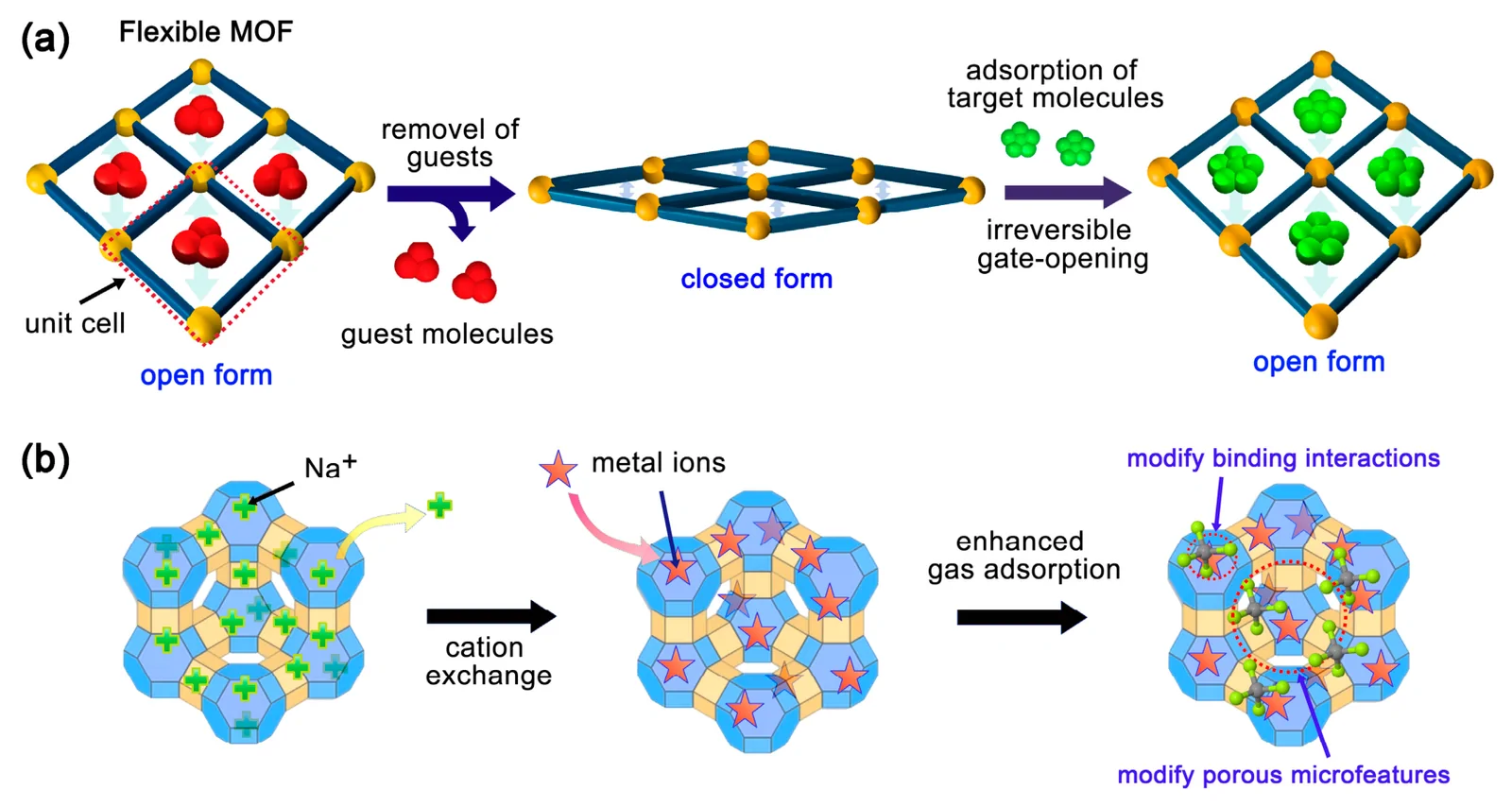
Schematic illustrations of (**a**) the gate-opening effect in MOFs. The framework, composed of yellow spheres (metal nodes) and blue rods (organic linkers), represents the MOF structure. Red and green clusters denote guest gas molecules. The arrows indicate the expansion or closed framework upon gas adsorption and desorption. (**b**) cation-exchange-induced enhancement of gas adsorption in zeolites. The cations (e.g., Na^+^, denoted as green crosses) in the framework are exchanged with metal ions (denoted as red stars), as indicated by the arrows, leading to enhanced adsorption of gases. Dashed cycles represent the modified nanofeatures of zeolite structure.

While precise pore size control enables molecular sieving, an orthogonal strategy lies in exploiting framework dynamics. Certain MOFs exhibit pronounced “gate-opening” or “breathing” behavior [42,43], where their frameworks undergo reversible structural transformations upon exposure to specific guest pressures or concentrations (Figure 3a). Such dynamic responses create ultra-strong confinement effects, significantly enhancing selectivity for target VFCs over smaller non-fluorinated competitors. This behavior may not only boost adsorption capacity but also establish a sharp threshold mechanism for sensing applications, where signal transduction occurs only beyond a critical VFC concentration. Beyond MOFs, HOFs and POCs may also exhibit dynamic behavior by virtue of their distinct structural construction. HOFs are intrinsically capable of guest-responsive structural transformations, including hydrogen-bond reformation and framework shrinkage/expansion [44]. POCs can undergo conformational changes that modulate cavity accessibility in response to external stimuli [45]. In contrast, other porous materials either completely lack dynamic structural adaptability or exhibit only limited, local conformational adjustments. This fundamental distinction positions dynamic frameworks and molecular cages as uniquely suited for applications requiring both high-fidelity VFC recognition and adaptive capture [46].

### 2.2. Modulation Strategies by Surface Chemistry

Surface chemistry could offer an additional dimension for tailoring VFC recognition. Purely physical confinement often yields insufficient selectivity for VFCs, particularly when target species share similar molecular dimensions or compete with background interferents. Surface modification could modulate the electronic environment of the pore interior, enabling stronger and more selective interactions. For most applications, adsorption is always based on three dominant interaction mechanisms. They are electrostatic effects (encompassing ion–quadrupole and dipole–induced dipole contributions for symmetric VFCs), fluorophilic affinity for fluorinated analogues, and hydrogen bonding for HFCs that possess C–H moieties. Surface modification can modulate the electronic environment of the pore interior, enabling stronger and more selective interactions through these pathways.

Open metal sites, i.e., coordinatively unsaturated metal centers, represent a particularly effective form of electrostatic interaction. Lewis acidic metal centers (e.g., Cu^2+^, Cr^3+^, Co^2+^) are capable of polarizing chemically inert VFCs via ion–quadrupole interactions [47,48], thereby markedly strengthening their binding affinity. The ability to tune open metal sites varies significantly across material classes.

MOFs are particularly well suited in this regard, offering a vast library of metal nodes that can be systematically varied to optimize binding strength. The density and accessibility of open metal sites can be finely tuned by tailoring linker architecture and adjusting activation protocols, including solvent exchange and vacuum-assisted thermal treatment [49,50].

In zeolitic and porous oxide frameworks, coordinatively unsaturated metal centers are inherently absent. Nonetheless, zeolites can generate localized electrostatic fields through the presence of extra-framework cations [51]. As shown in Figure 3b, the identity of the cations, such as Na^+^, can be exchanged, allowing tuning of the adsorption affinity toward specific molecules. For fully organic materials, including COFs, HOFs, and POCs, metal sites are not intrinsic to the framework. To introduce metal functionality, post-synthetic strategies, such as ion exchange, metal doping, or metalation, are required [52,53]. However, these approaches generally yield lower site densities and less uniform distribution compared to MOFs.

Besides open metal sites, heteroatom doping or fluorophilic functionalization is also widely applied for modulating surface chemistry. Heteroatom doping introduces non-metal elements (N, O, S, B, P) into carbonaceous or organic frameworks, altering local electron density and creating polar sites [54,55]. These sites can interact with VFCs through dipole–dipole or dipole-induced dipole interactions. However, precise control over dopant spatial distribution is not an easy task. Understanding of dopant–pore synergy and mitigation of performance degradation under humid conditions, where competitive adsorption with water vapor intervenes, are also unresolved challenges.

For fluorophilic functionalization, the introduction of fluorine-containing moieties provides unique electronic characteristics and engages in complementary electrostatic potentials with fluorinated guests. F–F interactions and dispersion forces contribute to the selective binding of VFCs over non-fluorinated competitors [56]. However, the synthesis of fluorinated porous materials often relies on expensive precursors and harsh conditions. The introduction of bulky fluorinated moieties tends to diminish the accessible pore volume and may occur at the expense of framework crystallinity in more pronounced cases.

### 2.3. Design Criteria for VFC Sensing and Capture

While traditional metrics, such as adsorption capacity, selectivity coefficients, and response kinetics, provide a necessary baseline, evaluating materials for VFC management requires a shift in perspective. Unlike CO_2_ or conventional VOCs, the capture and sensing of VFCs requires a distinct and stringent set of operational constraints, which include the pervasive interference of ambient humidity, regulatory detection thresholds extending into the sub-ppm range, and the potential generation of corrosive decomposition byproducts. These extreme conditions not only add experimental complexity but also may fundamentally change the usual design rules for adsorbents. Rethinking performance criteria and engineering strategies therefore becomes essential.

The performance requirements for capture and sensing diverge in some respects but converge on the need for robustness. Optimizing capacity and selectivity is one of the main issues for gas adsorption. Maximizing capacity often calls for larger pores or higher surface areas, which can inadvertently admit interfering species and compromise selectivity. Ultra-small pores, by contrast, offer high selectivity through molecular sieving but often suffer from slow diffusion and limited capacity. Hierarchical pore structures, in which larger transport channels converge upon smaller cages bearing high-affinity sites, present a viable resolution to this trade-off.

Another challenge lies in balancing the binding affinity of the gas adsorbents with their regenerability. If VFCs bind too strongly, thermal or vacuum-assisted desorption is required, raising both operational expense and the risk of material degradation. A promising alternative is dynamic frameworks that offer moderate binding but release guests upon a mild stimulus.

Sufficient stability under practical operating conditions is also essential, as highly sensitive sensors often rely on delicate electronic states or fragile porous architectures that degrade upon repeated cycling. Beyond intrinsic chemical and thermal stability, moisture resilience represents the definitive test for real-world applicability. The devices need to maintain robust performance under humid conditions, for example, at a relative humidity of 80%, which is a commonly adopted test condition for evaluating moisture tolerance in adsorption studies [57,58].

Overall, VFC management lies not in optimizing single parameters, but in balancing different constraints. While distinct porous platforms possess unique intrinsic advantages, the optimal design invariably lies in the strategic integration of multiple mechanisms. For instance, fluorinated MOFs incorporating open metal sites can simultaneously leverage electrostatic polarization from metal centers and fluorophilic affinity from functionalized linkers, achieving synergistic enhancement specifically for perfluorinated molecules [59]. Similarly, heteroatom-doped carbons with fluorinated surface groups offer a balanced approach for complex VFC streams by combining pore confinement with tailored surface chemistry [60]. These multi-modal integrated approaches, which integrate high capacity, superior selectivity, and stability, represent the most promising frontier for next-generation dual-functional VFC control materials.

## 3. Porous Materials for VFC Management

The design principles outlined above provide the foundation for deploying porous materials in VFC sensing and capture. However, evaluating material performance in these two domains demands fundamentally different analytical criteria. For sensing applications, the primary concern is the transduction of molecular recognition into detectable outputs. The choice of transduction mechanism determines the sensitivity and response characteristics. We therefore organize sensing platforms by signal readout to facilitate direct comparison of detection limits and response dynamics across different material systems. For capture applications, performance metrics such as capacity, selectivity, and working stability are strongly correlated with the physicochemical properties of the target VFC species. Each subclass (SF_6_, PFCs, HFCs, CFCs/HFOs, and NF_3_, etc.) with different polarizability, kinetic diameter, and molecular topology may present distinct challenges in molecular sieving and affinity modulation. Our discussion of capture is thus structured around VFC categories.

### 3.1. Porous Materials in VFC Sensing

Porous frameworks are attractive transducer materials for VFC sensing, benefiting from high surface areas and chemically tunable pore environments that facilitate both analyte adsorption and selective molecular recognition. Depending on the transduction pathway, the host–guest interactions can be monitored electrically, optically, or mechanically.

#### 3.1.1. Chemi-Resistive Transduction

Chemiresistive sensors translate gas adsorption into changes in electrical resistance or conductivity [61]. The primary recognition pathway for VFCs relies on converting Lewis acid-based interactions at the sensor surface into measurable resistance. The interaction between Lewis acidic sites and the localized negative charge on fluorine atoms modulates the carrier concentration in the transducer, producing a resistance change without requiring strong chemical bonding. The sensitivity and selectivity of such responses are therefore governed by the density and strength of surface Lewis acid sites, and the conductivity of the transducer.

Conductive MOFs and COFs offer structural tunability that distinguishes them from conventional oxides [62,63]. Lei et al. performed a DFT study on Zr-MOF-808 for SF_6_ and CF_4_ detection, predicting that adsorption-induced conductivity changes could enable gas identification [64]. The prediction relies on the accessible Zr(IV) Lewis acid sites within the MOF channels, where the high charge density of the metal center enhances electrostatic attraction toward fluorinated species. Although experimental validation remains lacking, recent reviews have systematically examined how pore structure, film morphology, and electrode configuration affect signal transduction and analyte diffusion in MOF- and COF-based chemiresistive sensors [65,66,67,68]. These emerging strategies, including oriented MOF thin films, hierarchical pore structures, and gradient-density sensing layers, offer potential for enhanced analyte accessibility and improved response kinetics, and may be extendable to VFC detection.

Metal oxides represent the most established class of chemiresistive materials, offering complementary strengths in thermal stability, long-term reliability, and established manufacturing processes [69]. Recent studies have demonstrated that even perfluorinated VFCs can generate measurable chemiresistive responses on metal oxide surfaces. Phansiri et al. employed dielectrophoretic assembly to fabricate SnO_2_ nanoparticle sensors and reported a progressive decline in conductance upon exposure to 1% CF_4_ in SF_6_, with the magnitude of the response [70]. Although the detection limit remains relatively high (10,000 ppm), this work provided direct experimental evidence that metal oxide chemiresistors are capable of detecting inert perfluorinated gases. The sensitivity was attributed to the surface Sn^4+^ sites, which serve as Lewis acids to interact with fluorine lone pairs, depleting electrons from the n-type SnO_2_ and increasing resistance [71].

A substantial improvement in detection limit (7 ppb) was recently achieved by Meng et al. using an N-doped SnO_2_ sensor, which exhibited sensitivity toward several F-type gases (SF_6_, C_2_F_6_, and C_2_H_2_F_4_) [72]. The nearly three-order-of-magnitude enhancement over undoped SnO_2_ provides a clear illustration of how defect engineering modulates surface electronic structure. For the undoped SnO_2_ sensor, the responses to SF_6_, C_2_F_6_, and C_2_H_2_F_4_ were comparable in magnitude, with response and recovery times following the order SF_6_ < C_2_F_6_ < C_2_H_2_F_4_. This trend correlates with the molecular size and adsorption/desorption kinetics. Upon N doping, the response patterns diverged substantially. The R_a_/R_g_ value for C_2_F_6_ became nearly six times higher than that for the other two gases, while response times (T_res_) for all three species converged to similar values. The recovery time (T_rec_) for C_2_F_6_ increased markedly relative to SF_6_ and C_2_H_2_F_4_, suggesting that N doping introduces adsorption sites with enhanced affinity toward C_2_F_6_. These results point to a more complex mechanism than simple electrostatic enhancement. The N dopant may alter surface defect distribution or create favorable adsorption geometries that preferentially stabilize C_2_F_6_ on the sensor surface. The enhancement achieved through N doping exemplifies a broader defect engineering paradigm, which is particularly valuable for amplifying weak signal responses from inert perfluorinated gases by modulating surface electronic structure and creating additional active sites.

**Figure 4:**
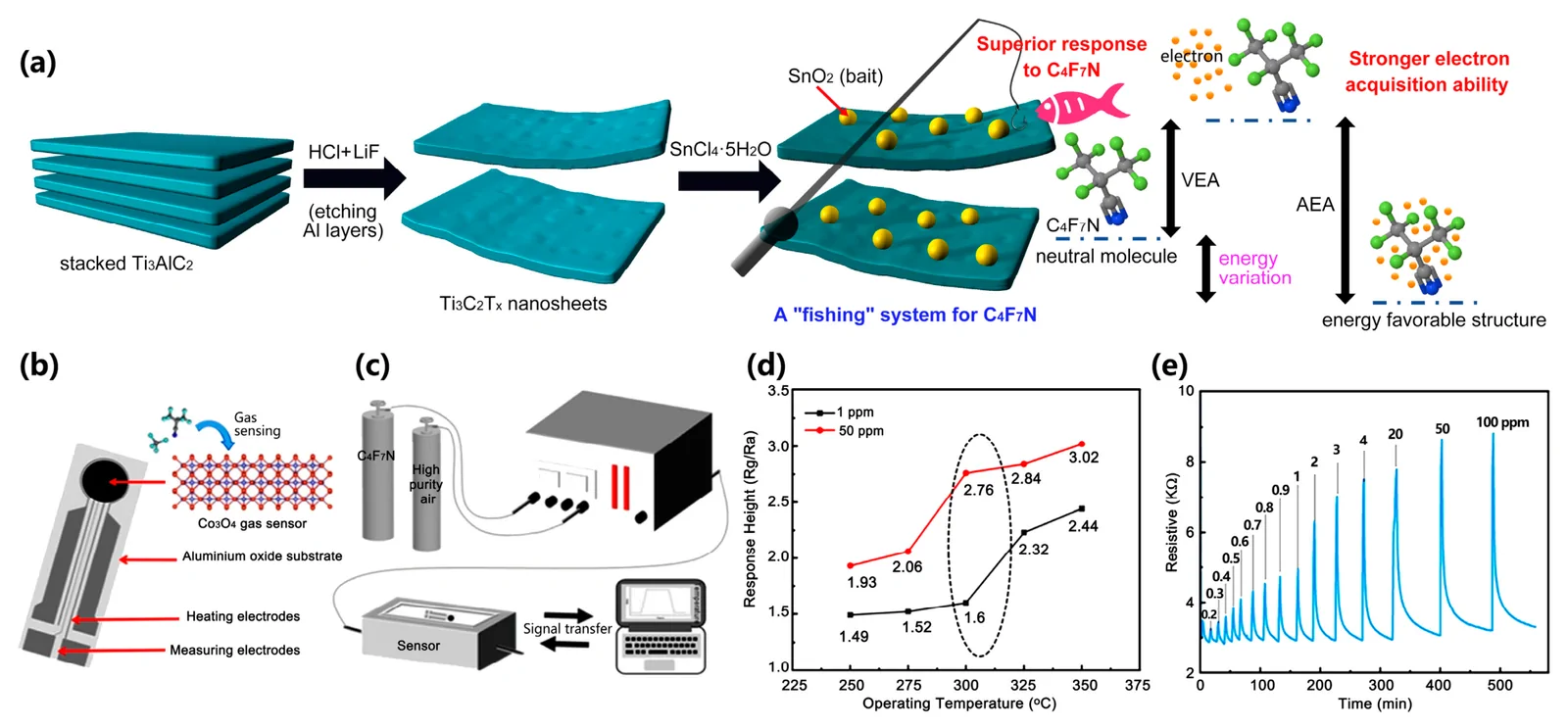
(**a**) Schematic illustration of the SnO_2_-modified Ti_3_C_2_T_x_ “fishing” system for C_4_F_7_N detection. The system is prepared by the etching of Al layers in Ti_3_AlC_2_ and modification with SnO_2_ (as “bait”). The enhanced response toward C_4_F_7_N (as “red fish”) is attributed to its strong electron-acquisition capability, as rationalized by the definitions of vertical electron affinity (VEA), adiabatic electron affinity (AEA), and the energy variation diagram (left part). The dashed lines with different patterns represent different electronic states. (**b**) Schematic diagram of the Co_3_O_4_-based gas sensor. (**c**) Dynamic sensing response analysis platform. (**d**) Response of the Co_3_O_4_ sensor toward 1 and 50 ppm of C_4_F_7_N at different operating temperatures. The dashed oval box marks the optimal sensing condition. (**e**) Transient response curves of the Co_3_O_4_ sensor to different concentrations of C_4_F_7_N (operation at 300 °C). Adapted with permission from [75]. Copyright 2025 American Chemical Society.

As a sustainable alternative to SF_6_, C_4_F_7_N has recently gained attention with growing interest in its detection. Wu et al. first demonstrated SnO_2_ nanoparticle-based sensors for C_4_F_7_N at 275 °C [73]. The high operating temperature reflects the activation energy required for C_4_F_7_N to overcome the surface adsorption barrier. This limitation was subsequently addressed by the same group through the design of SnO_2_/Ti_3_C_2_T_x_ MXene nanocomposites [74]. The design follows a “fishing” strategy (Figure 4a). SnO_2_ nanoparticles act as “baits” that provide strong Lewis acid sites to interact with the electronegative fluorine atoms of C_4_F_7_N, while the high-conductivity Ti_3_C_2_T_x_ MXene serves as the “fishing rod” or conductive platform for signal transduction [74]. This functional configuration enabled room-temperature operation. The response of the optimized M-S_40_ sensor to varying C_4_F_7_N concentrations (10–45 ppm) showed a sensitivity improvement of 460% compared to pristine MXene. The SnO_2_/MXene heterojunction enhances the resistance change upon analyte binding through synergistic charge transfer between the two components.

Most recently, Xiao et al. reported Co_3_O_4_ nanoparticle sensors for C_4_F_7_N leakage detection [75] (Figure 4b–e). As a p-type semiconductor, Co_3_O_4_ operates through a distinct mechanism from n-type SnO_2_. Notably, n-type materials typically show increased resistance upon VFC adsorption due to electron depletion, while p-type materials exhibit the opposite trend through hole accumulation. Oxygen adsorption on Co_3_O_4_ extracts electrons from the valence band, establishing a hole accumulation layer at the surface. Upon exposure to C_4_F_7_N, the fluorine atoms interact with surface oxygen species or oxygen vacancies, altering the hole concentration and thereby modulating the measured resistance. The sensor achieved a detection limit of 0.15 ppm at 300 °C, with exceptional selectivity and repeatability, highlighting the potential of Co_3_O_4_ for practical leakage monitoring.

Sensor stability and long-term reliability are critical for practical deployment, yet they remain underexplored in VFC sensing. However, careful material design has yielded exceptional operational stability in related chemiresistive systems [76]. For example, a porphyrin-based COF sensor for NO_2_ detection exhibited a minimal response variation of 3% over six days and maintained performance for over 75 days [77]. For VFC sensors, the SnO_2_/MXene and Co_3_O_4_ systems discussed above have demonstrated good repeatability over multiple cycles. These achievements suggest that strategies such as post-synthetic stabilization, protective coating, and optimized film fabrication could significantly enhance the durability of VFC sensors.

These studies, from conductive MOF/COF-based theoretical explorations to metal oxides and rationally designed composites, illustrate how structural design and mechanistic understanding drive the evolution of chemiresistive sensors for VFCs. However, the overall body of work on VFC sensing remains far less explored than capture. Most studies have focused on SF_6_, while reports on NF_3_, PFCs, and HFCs are scarce. Many reported detection limits are also far above practical regulatory targets.

#### 3.1.2. Optical and Gravimetric Transduction

Beyond chemiresistive transduction, optical and gravimetric methods offer alternative routes for VFC detection. Both approaches rely on physical changes induced by guest adsorption rather than charge transfer. Optical sensing monitors variations in refractive index, absorption, or fluorescence for optical sensing, and mass loading or damping for gravimetric detection, rather than charge transfer.

Most VFCs are inherently weakly absorbing across the ultraviolet-visible (UV-vis) spectrum, which precludes straightforward optical readout. A diverse array of materials has been tailored for this application, encompassing organic small molecules, carbon-based nanomaterials, lanthanide coordination complexes, luminescent MOFs, COFs, and hybrid composite systems [78,79,80]. However, the majority of these efforts target PFAS in aqueous media rather than volatile species.

For example, Han et al. synthesized eight Eu-MOFs using a single linker, 2,5-furandicarboxylic acid (H_2_FDA), with systematically varied coordination symmetry [81]. MOFs with lower symmetry (Eu-FDA-5/6/7/8) exhibited significantly higher quenching efficiency than their high-symmetry counterparts, attributed to enhanced energy-level splitting that promotes electron transfer to perfluorooctanoic acid (PFOA). In this system, the MOF serves as the energy/electron donor upon photoexcitation via the linker-to-Eu energy transfer. The asymmetric coordination environment enhances the Stark splitting of the Eu^3+^ 4f energy levels and facilitates photoinduced electron transfer (PET) from the excited MOF to PFOA. The consequent depletion of excited-state electrons leads to a turn-off luminescence response, with the quenching degree directly correlating with PFOA concentration. This work establishes coordination geometry as a decisive design parameter, distinct from pore dimensions or surface area, in enhancing sensing performance. The same group also developed millimeter-scale single crystals of ITHD(Zn), a Zn-based MOF [Zn_6_(BTB)_4_(bipy)_3_; BTB = benzene-1,3,5-tribenzoate, bipy = 4,4′-bipyridine], enabling reusable luminescent sensing of PFAS [82]. Unlike conventional MOF powders requiring ultrasonic dispersion, these large crystals can be directly immersed, retrieved, and reused over ten cycles without performance loss, with specific host–guest interactions inducing luminescence enhancement rather than quenching.

Although PFAS and VFCs differ markedly in molecular structure and physicochemical properties, both lack intrinsic optical activity for direct fluorescence detection. The design principles established for PFAS detection offer a valuable conceptual reference for gaseous VFC sensing.

**Figure 5:**
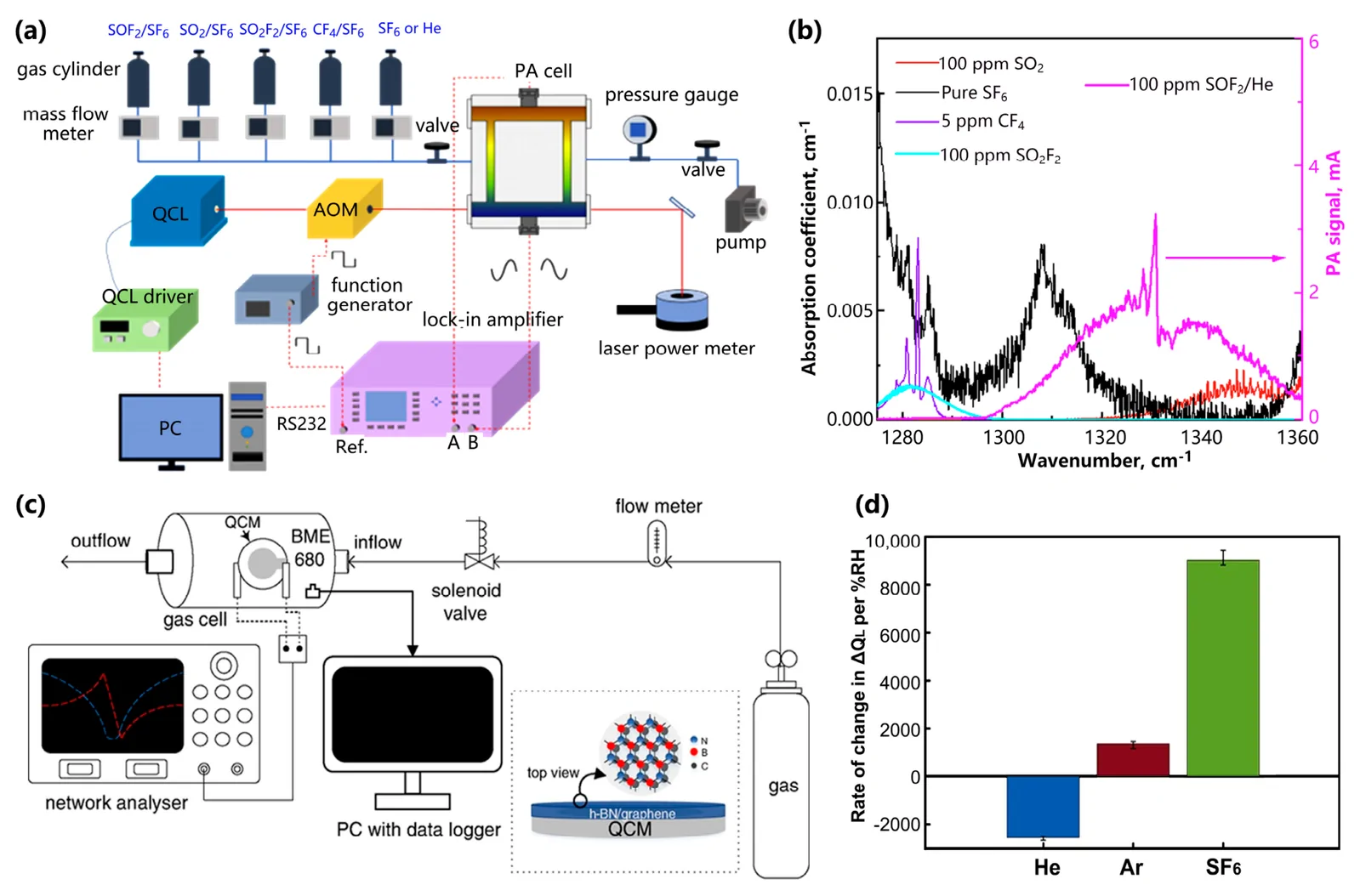
(**a**) PAS experimental setup for SF_6_ decomposition by-product detection. The blue block labeled “QCL” denoted the quantum cascade laser. The green block denotes the QCL driver. The purple block denotes the lock-in amplifier. The yellow block labeled “AOM” denoted the acousto-optic modulator. The center blue block denotes the function generator. The light blue square labeled “PC” denoted the personal computer. The blue short cylinder denotes the laser power meter. The abbreviation “Ref.” refers to “external reference”. The labels A, B indicate input. The red solid line represents optical path. The red dashed line represents electrical path. The blue solid line represents gas path. RS232 indicates serial port. (**b**) Simulated absorption spectra of SF_6_, SO_2_F_2_, SO_2_, and CF_4_. Adapted from Ref. [84]. (**c**) Schematic of the Hg-QCM experimental setup for electronic gas flow sensing. (**d**) Hg-QCM’s rate of change in ΔQ_L_ per %RH for typical electronic gases, He, Ar, and SF_6_, at a flow rate of 130 cc·min^−1^. Adapted from Ref. [91].

Photoacoustic spectroscopy (PAS) offers a distinct optical transduction mechanism that does not rely on porous materials for signal generation. The acoustic wave is induced by direct optical absorption of the analyte. This technique is inherently suited to VFCs that exhibit strong mid-infrared C–F stretching vibrations [83]. Wei et al. employed an external cavity quantum cascade laser to exploit these absorption fingerprints, achieving simultaneous detection of SO_2_F_2_, SOF_2_, CF_4_, and SO_2_ (Figure 5a,b), achieving a detection limit of 10.3 ppb for CF_4_ [84]. Ji et al. later replaced the mid-infrared laser with a low-cost blackbody radiation source, achieving an SF_6_ detection limit of 408.8 ppb (6.83 ppb with signal averaging) [85]. While not reliant on porous transducers, PAS provides a complementary optical route for VFC detection where high sensitivity and multicomponent analysis are required.

Gravimetric transduction, typically based on quartz crystal microbalances (QCMs), detects adsorption through frequency shifts proportional to adsorbed mass. The sensing performance, particularly in terms of selectivity and sensitivity, depends critically on the tailored design of the electrode surface coating [86,87]. The coating must provide accessible recognition sites that maximize mass-loading effects for target molecules over interferents. However, perfluorinated VFCs generate only weak, non-covalent interactions with typical QCM coatings, yielding mass changes below the detection threshold. Therefore, most successful QCM reports focus on decomposition products as indirect indicators of VFC leakage, rather than on direct VFC detection. This is because these byproducts are generally more chemically reactive than the parent VFCs and thus are easier to detect through mass changes. Yang et al. employed NH_2_-MIL-101(Cr) as the QCM coating to detect hydrogen fluoride (HF), a decomposition product of SF_6_ and other fluorinated gases [88]. The amino-functionalized MOF offers high surface area and accessible pore channels, which may facilitate HF diffusion to the –NH_2_ sites and maximize the mass-loading effect. The hydrogen bonding between the –NH_2_ groups and HF molecules provides the recognition mechanism, achieving a detection limit of 500 ppb for anhydrous HF with short response times and good reversibility. This work demonstrated that with appropriate surface functionalization, QCM can effectively detect reactive fluorinated species through standard mass-loading transduction.

Beyond academic studies, commercial high-frequency QCM (HF-QCM) platforms have also emerged. For example, the HF-QCM technology from MS Detection, which integrates proprietary chemically sensitive coatings with engineered sensor array architectures, has been successfully translated into field-deployable systems for applications such as food contaminant screening and industrial safety monitoring [89,90].

To overcome the weak adsorption of inert VFCs on the QCM electrode, an alternative transduction pathway of damping rather than frequency shift has been explored. Leong et al. integrated a hexagonal boron nitride–graphene (hBN-graphene) monolayer heterostructure onto the QCM electrode (Figure 5c,d) [91]. This coating layer, characterized by a high elastic modulus and a minimized cross-sectional area, serves to reduce hydrodynamic drag while enhancing sensitivity to interfacial energy dissipation, thereby amplifying damping-mediated responses to gas exposure. Upon introduction of humidified SF_6_, the sensor displayed negligible frequency variation yet exhibited marked alterations in the loaded quality factor (ΔQ_L_), which scaled systematically with SF_6_ flow rate. This behavior is attributed to gas-flow-induced perturbations in motional resistance (R_m_) rather than mass-loading effects. Although the hBN-graphene coating lacks the three-dimensional porosity of MOFs or COFs, its atomically thin geometry enables a distinct transduction mechanism that overcomes the weak adsorption of perfluorinated VFCs. The study constitutes the first demonstration of SF_6_ detection via a QCM relying on damping phenomena. It confirms that even chemically inert VFCs can be detected with high sensitivity through mechanical dissipation when paired with appropriately tailored two-dimensional coatings.

By comparison, optical and gravimetric methods remain less explored for direct VFC sensing than chemiresistive approaches. While PFAS sensing has provided useful design principles for optical sensing of VFCs, translating these strategies to gaseous VFCs is challenging due to the much lower analyte density in the gas phase. PAS, though sensitive, relies on costly mid-infrared laser sources that limit portability for on-site use. Gravimetric methods are constrained by the weak physisorption of perfluorinated VFCs. Most successful QCM studies have focused on reactive decomposition products rather than the parent gases. A damping-mode QCM with 2D coatings has shown promise for SF_6_ detection, but this approach remains preliminary. Nevertheless, these methods offer distinctive capabilities (e.g., remote operation, optical readout without conductive materials, and mechanical detection without electrical functionality) that complement chemiresistive sensors [92]. Their continued development could be particularly valuable for scenarios where electrical signal transduction is impractical or for orthogonal validation of chemiresistive results.

#### 3.1.3. Catalytic Conversion-Based Sensing

Given the limitations of direct host–guest interactions for exceptionally inert VFCs, an alternative strategy, catalytic conversion, has emerged to enable their detection by transforming these gases into more reactive, detectable species.

A foundational patent described a compact catalytic conversion sensor specifically designed for fluorine-based special gases used in semiconductor manufacturing [93]. The device uses a noble metal catalyst (Pd and Pt) heated to 300–700 °C to oxidize NF_3_ to NO_2_. The resulting NO_2_ was subsequently quantified using an electrochemical sensor element. This configuration yields markedly higher conversion efficiency than thermal decomposition alone.

Powertech Labs has developed a portable handheld instrument for the on-site detection of SF_6_ decomposition products in gas-insulated electrical equipment [94]. The device contains a chamber packed with fine silica gel (100–120 mesh) heated to approximately 200 °C, which catalytically converts the primary SF_6_ decomposition products, SOF_2_ and SF_4_, into SO_2_ for downstream electrochemical detection. The silica gel serves not only as support but also as a porous scaffold for catalyst dispersion and gas diffusion. The system achieves a detection limit of 1 ppm for SOF_2_ and can be operated on energized equipment. This approach dramatically outpaces conventional methods, eliminating the need for weeks-long lab workflows.

These studies highlight the adaptability of catalytic conversion to diverse operational regimes for VFC detection, enabling trace-level quantification with real-time response. The emergence of commercial instruments based on this principle further attests to its technical maturity and industrial relevance. However, most current systems rely on conventional porous supports (silica gel, Al_2_O_3_) and require high operating temperatures (typically 200–700 °C). Future development could benefit from advanced porous materials as catalyst supports, where the synergistic effects of pore confinement, surface functionality, and active site dispersion may enable lower-temperature operation and expanded VFC coverage.

### 3.2. Selective Adsorption of Targeted VFCs

Gas adsorption represents a complementary function to sensing in VFC management. While sensing focuses on the transduction of molecular recognition into measurable signals, capture aims to adsorb VFCs from gas streams with high capacity and selectivity. The relevant performance is governed by the same fundamental design parameters (pore size, surface functionality, and framework flexibility), but it is optimized toward different objectives. The following sections evaluate capture performance across the major VFC subclasses of SF_6_, NF_3_, PFCs, HFCs, and CFCs/HFOs, each with distinct molecular characteristics and industrial contexts.

#### 3.2.1. Trapping of Electron-Industrial Gases (SF_6_ and NF_3_)

SF_6_ is the most potent greenhouse gas among VFCs, with a GWP value of 23,500 and an atmospheric lifetime exceeding 3000 years [95]. It possesses an octahedral symmetry structure with a kinetic diameter of ~5.5 Å and a negligible dipole moment. NF_3_ exhibits comparable characteristics, with a GWP of 16,100, an atmospheric lifetime of ~740 years, and a kinetic diameter of ~4.5 Å. Both lack a significant dipole moment and rely on polarizability-based interactions for selective adsorption. Table 1 summarizes representative porous materials for SF_6_ and NF_3_ capture, highlighting their structural features and adsorption performance.

Among MOF-based adsorbents, the DMOF-Cl series reported by Nie et al. provides a systematic demonstration of pore size modulation through linker halogenation [96]. Progressive chlorination of the organic linkers contracted the pore aperture from 8.9 Å (DMOF-1) to 6.5 Å (DMOF-4Cl), accompanied by concurrent modulation of surface electrostatic potential. The resultant DMOF-4Cl achieved an SF_6_ capacity of 2.0 mmol·g^−1^ at 10 kPa and an SF_6_/N_2_ selectivity significantly exceeding that of the non-chlorinated counterpart. Breakthrough experiments confirmed that high-purity SF_6_ (>99.1%) was recoverable in a single cycle. This work illustrates how sub-angstrom pore control via linker functionalization can simultaneously enhance capacity and selectivity. The trade-off between capacity and selectivity is further illustrated by comparing UiO-66 and UiO-67 [98]. The larger pores of UiO-67 yield higher SF_6_ uptake than UiO-66, but at the cost of reduced selectivity.

**Figure 6:**
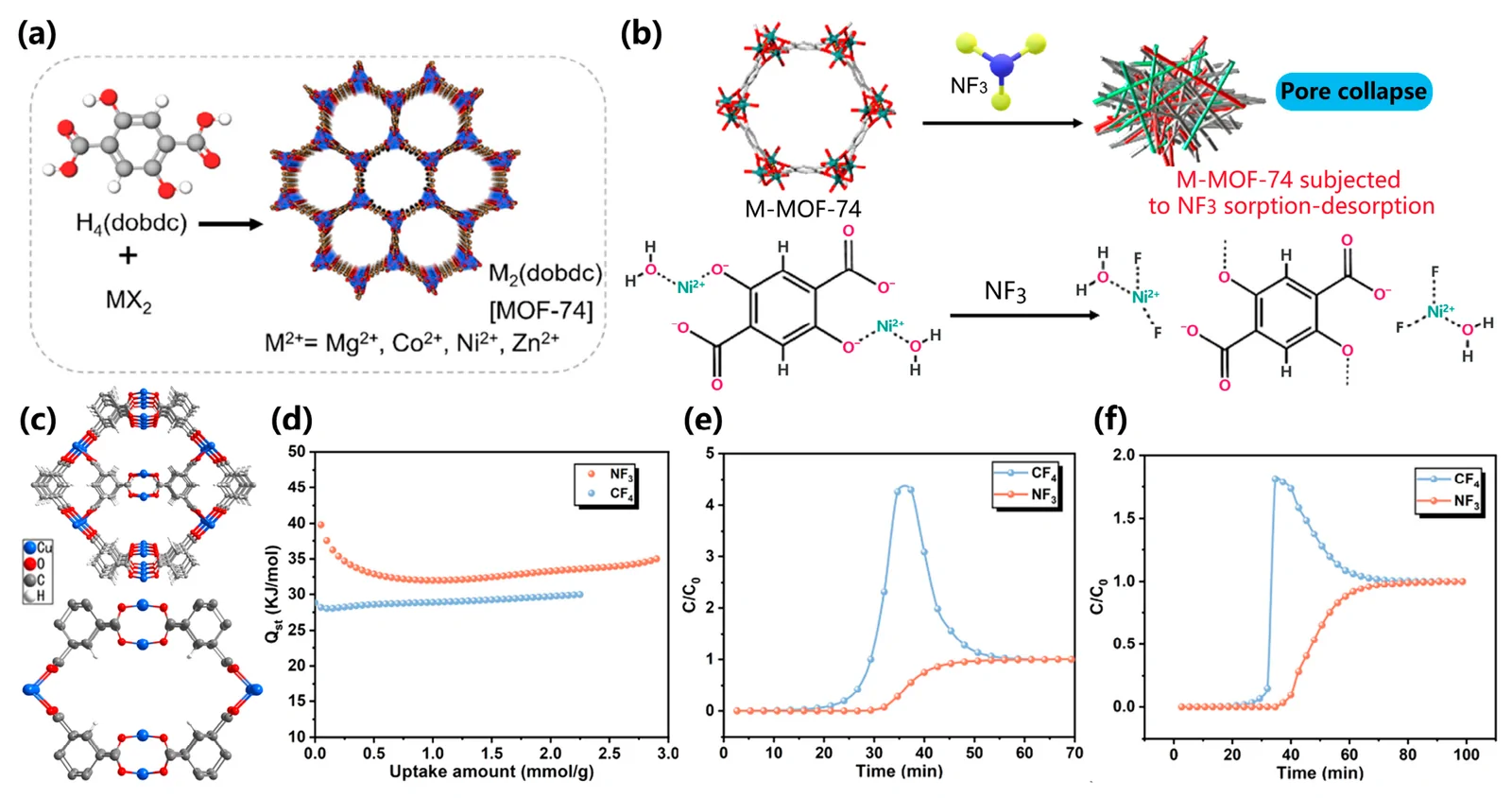
(**a**) Crystal and pore structures of MOF-74 with 1D hexagonal channels (in the dashed box), which is assembled from H_4_(dobdc) and metal salts (MX_2_). (**b**) The proposed NF_3_ destructive sorption mechanism on M-MOF-74. The porous framework collapses into a disordered structure upon NF_3_ adsorption. Adapted from Ref. [99]. (**c**) Channel structure of ATC-Cu with adjacent copper paddle-wheel and multiple alkane hydrogen atoms pointing to the pore. (**d**) Isosteric heats of adsorption (Q_st_) of NF_3_ (brown dot) and CF_4_ (blue dot). Experimental breakthrough curves for NF_3_/CF_4_ (*v*:*v*): (**e**) 1:1 and (**f**) (9:1). Adapted with permission from [101]. Copyright 2025 American Chemical Society.

For NF_3_ capture, the contrast between irreversible chemisorption (Co-MOF-74) and reversible physisorption (CALF-20, ATC-Cu) highlights a fundamental design choice between high-capacity one-time removal and cyclic operation [99,100,101]. Co-MOF-74 exploits open metal sites for strong electrostatic interactions, achieving a high NF_3_/N_2_ selectivity of 299.6 and notable uptake, at the cost of irreversible uptake and framework degradation (Figure 6a,b) [99]. In contrast, CALF-20 employs precise molecular sieving with 4.8 Å pores, achieving a record NF_3_/CF_4_ uptake ratio of 41.1 at 298 K [100]. Its stability is evidenced by a <2% capacity loss over five cycles and retained crystallinity. This sieving effect is supported by DFT calculations showing significantly higher diffusion barriers for CF_4_ compared to NF_3_, aligning with their kinetic diameters (4.8 Å vs. 4.5 Å). Fan et al. designed an MOF-based NF_3_ nanotrap (ATC-Cu) that operates via enhanced physical adsorption (Figure 6c–f) [101]. The material was synthesized under hydrothermal conditions from the ligand H_4_ATC (1,3,5,7-adamantanetetracarboxylic acid) and Cu(NO_3_)_2_·3H_2_O. Within the framework, the ATC linker serves as a tetrahedral node connected by four Cu paddle-wheel secondary building units, thereby generating a three-dimensional structure intersected by rectangular channels (4.43 × 5.39 Å^2^). The adjacent coordinatively unsaturated metal sites produce overlapping electric fields, which confer a strong localized affinity for NF_3_. As a result, ATC-Cu delivers high NF_3_ capacity and pronounced NF_3_/CF_4_ selectivity.

Zhang et al. reported an aluminum-based porphyrin MOF (Al-PMOF) featuring ultramicropores and polar Al–O clusters on the pore surfaces [102]. These polar sites enhance adsorption selectivity toward NF_3_ and SF_6_ through strong induced polarization interactions. At 298 K and 1.0 bar, Al-PMOF achieved adsorption capacities of 3.00 and 6.15 mmol·g^−1^ for NF_3_ and SF_6_, respectively. IAST calculations reveal an SF_6_/N_2_ selectivity of 581, and fixed-bed breakthrough experiments further validate its dynamic separation performance.

Among zeolites, cation exchange provides a versatile route for tuning adsorption performance. While the parent Na-Y shows only moderate SF_6_ affinity, K^+^-exchanged Y zeolite (K-Y) achieves an SF_6_ uptake of 2.20 mmol·g^−1^ at 0.1 bar and a dynamic SF_6_/N_2_ selectivity of 97.0 [103]. The GCMC simulations reveal that the six fluorine atoms of SF_6_ anchor to K^+^ cations through multiple cation-induced dipole interactions. Beyond cation exchange, heteroatom substitution in MFI frameworks (e.g., W^6+^) provides an additional route to enhance electrostatic polarization, improving both SF_6_ and NF_3_ selectivities [104]. Hierarchical structuring may further improve adsorption kinetics, as demonstrated by MFI-2, where the introduction of mesoporosity facilitates SF_6_ transport within the microporous framework and enables rapid adsorption–desorption cycling [117].

Carbon adsorbents provide a scalable and cost-effective platform. The dominant design parameter is ultramicropore engineering to achieve pore sizes closely matching the kinetic diameter of the target gas. Recent studies have demonstrated that precise pore control in the 0.5–0.9 nm range enables high SF_6_/N_2_ selectivities (up to 683.9) and NF_3_/N_2_ selectivities (up to 61.4) across diverse carbon precursors [105,106,107,108,109,110,111]. PVDF-derived carbons provide an additional benefit through in situ surface polarity from residual O/F groups, enabling efficient desorption at room temperature with >99.9% purity [109].

COF-based adsorbents have been explored through both computational and experimental approaches. Zhao et al. theoretically investigated the effect of heteroatom (N, O) functionalization on the SF_6_/N_2_ separation performance of COF-637 using GCMC simulations and DFT calculations [112]. At 298 K and 1 bar, the heterocyclic COF-2O and COF-2N exhibited SF_6_/N_2_ selectivities of 400.79 and 353.57, significantly outperforming the pristine COF-2C (177.24). Mechanistic analysis reveals that the introduction of heteroatoms may enable multiple host–guest interactions between SF_6_ and the framework.

**Figure 7:**
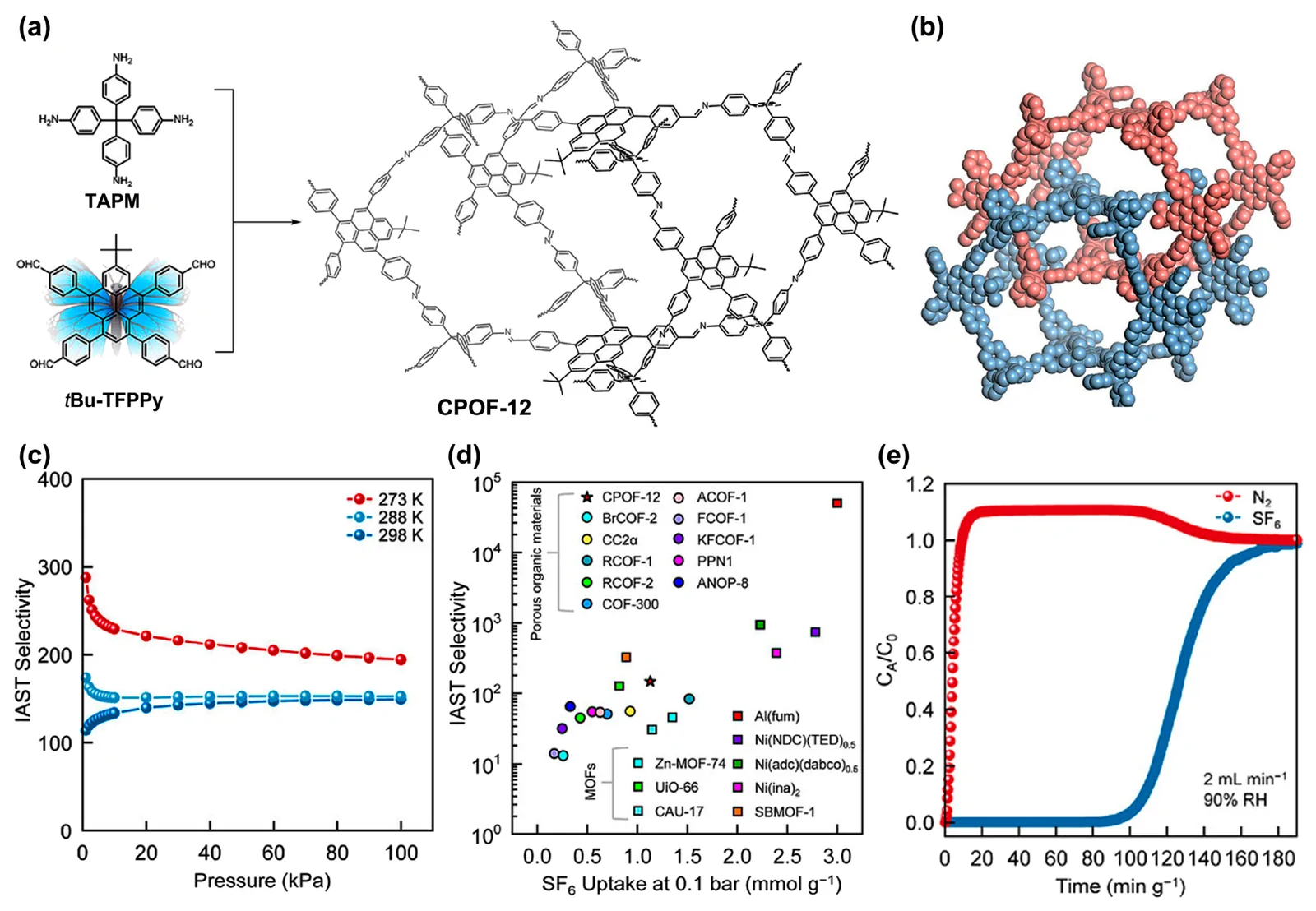
(**a**) Schematic illustration of CPOF-12 construction through an aldehyde–amine condensation reaction based on tetra(4-aminophenyl)methane (TAPM) and the butterfly-shaped 7-tert-butyl-1,3,5,9-tetrakis(4-formylphenyl)pyrene (*t*Bu-TFPPy) monomer, denoted by a blue butterfly mark The two tetrahedral building units are partially interwoven, representing the interpenetrated framework of CPOF-12, with *tert*-butyl groups extending into the pores. (**b**) Structural representation of the twofold interpenetrating framework (shown in red and blue) of CPOF-12. (**c**) The IAST selectivity curves of the SF_6_/N_2_ mixture for CPOF-12 at 273 K, 288 K, and 298 K, represented by red, light blue, and blue curves, respectively. (**d**) Comparison of the IAST selectivity and SF_6_ uptake (at 298 K and 0.1 bar) of CPOF-12 (star symbol) with other reported porous materials (square symbols). (**e**) Experimental breakthrough curve for the SF_6_/N_2_ mixture (10/90, *v*/*v*) on CPOF-12 under humid conditions (RH = 90%) with a total inlet flow rate of 2.0 mL min^−1^. The red and blue dotted curves represent the effluent concentrations of N_2_ and SF_6_, respectively. Adapted with permission from [114]. Copyright 2026 John Wiley & Sons, Inc.

Experimentally, RCOF-1 achieved an SF_6_ capacity of 4.13 mmol·g^−1^ with a selectivity of 125 at 273 K through optimized pore size (~0.9 nm) [113]. More recently, the synthesis of a three-dimensional COF (CPOF-12) provided experimental validation of precise aperture control for SF_6_/N_2_ separation [114]. CPOF-12 is constructed from tetrahedral building units with *tert*-butyl groups protruding into the pores (Figure 7). These bulky substituents narrow the pore aperture to 0.59 nm, closely matching the kinetic diameter of SF_6_. The structure also creates a hydrophobic, fluorophilic microenvironment that enhances C–H···F interactions with adsorbed SF_6_ molecules. The combined effects lead to an SF_6_/N_2_ selectivity of 149.4 [114], representing an important step from simulation-based understanding toward experimentally validated COF adsorbents.

Porous organic cages (POCs) have also been explored for SF_6_ capture through distinct synthetic strategies and structural designs. Early studies by Hasell et al. on imine-based cages demonstrated that CC3α, synthesized via Schiff base condensation of 1,3,5-triformylbenzene and 1,2-diaminocyclohexane, exhibits notable SF_6_/N_2_ separation performance [115]. The cage molecules pack in a window-to-window arrangement, forming a diamond topology, which contributes to its structural stability. Despite having pore apertures (3.6 Å) smaller than the kinetic diameter of SF_6_ (5.5 Å), the material achieves exceptional selectivity through cooperative diffusion and structural rearrangements of the molecular crystals. Breakthrough experiments confirmed that CC3α is effective for SF_6_/N_2_ separation with a selectivity of 76.5 at 298 K [116]. More recently, Yang et al. reported a tetrazine-based POC (TC1) featuring a rigid tetrahedral structure, prepared via a one-pot nucleophilic aromatic substitution (SNAr) reaction between 3,6-dichloro-1,2,4,5-tetrazine and a triazine-based triphenol building block [116]. TC1 exhibits high porosity, with a BET surface area of 1157 m^2^ g^−1^, and good chemical stability, surpassing most POCs formed through dynamic covalent chemistry. Its well-defined, electron-deficient cage cavity enables efficient SF_6_/N_2_ separation, showing the highest SF_6_ adsorption capacity and selectivity among all reported porous molecular materials [116].

The five classes of adsorbents discussed above differ markedly in their practical applicability and design constraints. The comparisons indicate that no single platform simultaneously maximizes selectivity, capacity, stability, and scalability. MOFs achieve high selectivity through rational pore engineering, but this structural precision is accompanied by complex synthesis and, for some frameworks, limited moisture stability. Zeolites offer fewer structural variables than MOFs through cation-exchange chemistry but compensate with greater industrial maturity and hydrothermal stability. Carbon adsorbents rely primarily on physical pore-size control rather than chemical functionality to achieve competitive selectivities at lower cost. COFs offer greater chemical tailorability than carbons, with covalent linkages that can improve stability over some MOFs, though their robustness generally remains below that of carbon-based materials. POCs exploit discrete molecular cavities and peripheral functionalization for precise recognition, yet both classes face challenges in scalable synthesis.

#### 3.2.2. Recovery of Refrigerants (PFCs, HFCs, CFCs, HFOs)

The refrigerant family encompasses PFCs, HFCs, CFCs, and HFOs, a diverse group spanning multiple generations of industrial gases [118,119]. Unlike SF_6_ and NF_3_, where high polarizability provides a handle for electrostatic capture, these refrigerants present separation challenges that depend on molecular topology: subtle differences in kinetic diameters for PFCs, dipole moments for HFCs and CFCs, and C=C double bonds for HFOs. The design strategy accordingly shifts from polarizability exploitation to molecular topology discrimination. Table 2 summarizes different porous materials for refrigerant capture, organized by gas family and key structural features.

##### Perfluorocarbons (PFCs)

PFCs, including CF_4_, C_2_F_6_, and C_3_F_8_, are characterized by fully fluorinated structures, negligible polarizability, and the absence of permanent dipole moments. Selective capture depends on size-selective confinement exploiting subtle differences in kinetic diameters (CF_4_ ~4.8 Å, C_2_F_6_ ~5.7 Å, C_3_F_8_ ~6.3 Å). It is also often combined with fluorophilic pore surfaces to promote favorable F–F interactions.

**Figure 8:**
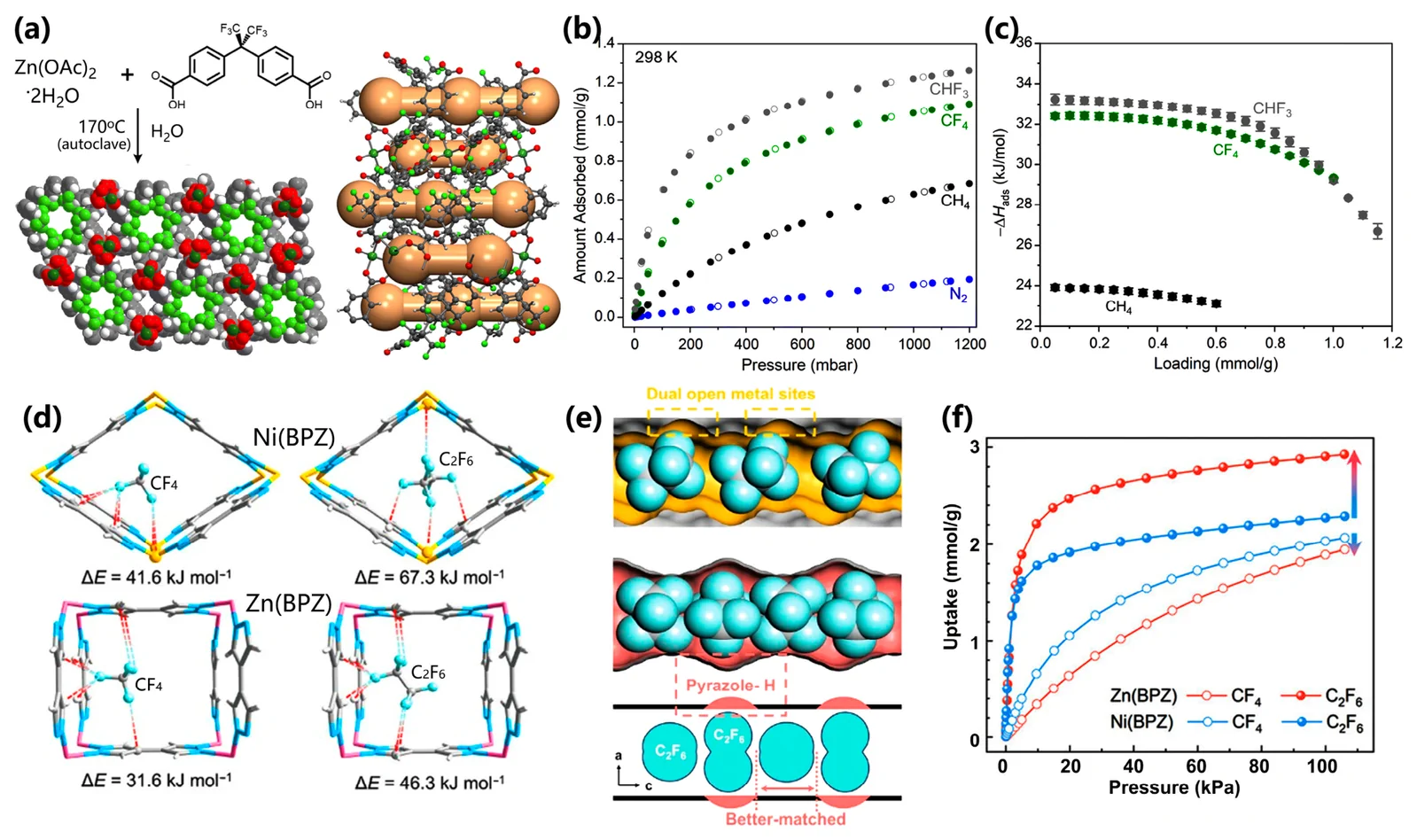
(**a**) Synthesis and solid-state structure of Zn(fba) and cavity plot depicting the dimensions of the arene-lined channels in Zn(fba). A space-filling model (**left**) is used wherein the van der Waals radii of atoms are depicted. Grey, white, red, light green, and dark green spheres correspond to carbon, hydrogen, oxygen, fluorine, and zinc atoms, respectively. Cavity plot (**right**) depicting the dimensions of the arene-lined channels in Zn(fba). These channels (brown) consist of spherical voids separated by narrow cylindrical windows. (**b**) Adsorption isotherm data for CHF_3_ (grey dots), CF_4_ (green dots), CH_4_ (black dots), and N_2_ (blue dots) in Zn(fba) at 298 K. (**c**) Differential enthalpy of adsorption as a function of loading for CHF_3_ (grey dots), CF_4_ (green dots), and CH_4_ (black dots). Adapted from Ref. [121]. (**d**) Preferential binding sites and binding energies simulated by DFT-D for CF_4_ and C_2_F_6_ in Ni(BPZ) and Zn(BPZ). The red dashed lines indicate multiple van der Waals interactions within the frameworks. (**e**) Snapshots of multi-C_2_F_6_ loading simulation results in the Ni(BPZ) and Zn(BPZ) channels, respectively. The dual open metal sites (yellow parts) in Ni(BPZ) channel provide strong binding affinity toward C_2_F_6_, but their distribution is not optimal for guest molecular stacking. The pyrazole-H groups (red parts) in Zn(BPZ) channel are more compatible with the C_2_F_6_ size, enabling higher space utilization. (**f**) The single-component adsorption isotherms for CF_4_ and C_2_F_6_ in Ni(BPZ) and Zn(BPZ) at 298 K. The arrows indicate that both materials prefer C_2_F_6_ adsorption over CF_4_. Adapted from Ref. [124].

The capture of CF_4_ requires pore sizes that accommodate the smallest PFC molecule while excluding N_2_. Ultramicropores with low-polarity surfaces have proven effective. Wan et al. designed a nickel-based MOF, Ni(ADC)(DABCO)_0.5_, featuring anthracene-ring-functionalized for CF_4_/N_2_ separation [120]. The material combines Ni_2_(COO)_4_ paddlewheel units connected by anthracene-based dicarboxylate (ADC) linkers and pillared by DABCO ligands. The resultant three-dimensional framework possesses nanospace pores and a low-polarity surface, which achieves a CF_4_ uptake of 0.52 mmol·g^−1^ at 0.1 bar and a CF_4_/N_2_ selectivity of 23.0. DFT analysis attributes this affinity to short F–π contact distances between fluorine atoms and the anthracene-functionalized pore walls [120]. Whitehead et al. extended this fluorophilic design by incorporating –CF_3_ groups as pore linings in Zn(fba), a hydrophobic MOF with 6.0 Å channels that achieves the highest CF_4_/N_2_ selectivity among water-stable sorbents (Figure 8a–c) [121]. This substitution of aromatic C–H with C–F groups enhances the fluorophilicity of the pore surface, promoting favorable F–F interactions with perfluorinated guests. Tian et al. further amplified this design principle by constructing isostructural imine cages with perfluorinated side chains (F-cage), where the complete fluorination of the cage exterior creates a dense array of F–F interaction sites [122]. The resulting c-C_4_F_8_/N_2_ selectivity (4385) far exceeds that of partially fluorinated or non-fluorinated analogues, demonstrating that the density of fluorophilic surface functionality correlates directly with recognition selectivity for perfluorinated species.

In semiconductor manufacturing, CF_4_ and C_2_F_6_ are both widely used perfluorocarbons, but CF_4_ is often recycled as a high-purity electronic-grade gas while C_2_F_6_ is present as a low-concentration impurity [139]. The separation of C_2_F_6_ from CF_4_ is particularly challenging due to their chemical inertness and structural similarity. Xu et al. addressed this challenge through a divergent adsorption regulation strategy using two isostructural MOFs [124], Ni(BPZ) and Zn(BPZ) (Figure 8d–f). In Ni(BPZ), the Ni^2+^ center leaves an open metal site after activation, whereas the Zn^2+^ center in Zn(BPZ) is coordinatively saturated. This subtle metal-node variation, combined with a change in channel geometry (rectangular in Ni(BPZ) vs. square in Zn(BPZ)), fundamentally alters the host–guest interaction landscape. As a result, CF_4_ showed reduced affinity in Zn(BPZ) due to the absence of strong binding sites. In contrast, C_2_F_6_ exhibits markedly denser packing within the Zn(BPZ) framework, owing to weaker net guest–host interactions from the pyrazole-H groups. The features enable unprecedented low-pressure C_2_F_6_ uptake with exceptional C_2_F_6_/CF_4_ selectivity [124].

The separation of C_3_F_8_ typically involves either removing it as a trace impurity from CF_4_ or C_2_F_6_ streams or purifying it from the closely similar hexafluoropropylene (C_3_F_6_). A straightforward approach to C_3_F_6_/C_3_F_8_ separation is to create pore apertures that allow the smaller C_3_F_6_ to enter, while excluding the larger C_3_F_8_. Zheng et al. developed a fluorinated cage-like MOF, Zn-bzc-CF_3_ (bzc: benzimidazole-based carboxylate ligand), by incorporating –CF_3_ groups into the pore windows of the parent MOF [125]. This fluorination strategy achieved ideal molecular sieving for C_3_F_6_/C_3_F_8_ separation, yielding C_3_F_8_ at a purity exceeding 99.9%. DFT calculations confirmed that the –CF_3_ groups can participate in multiple F···F and F···H–C interactions with adsorbed C_3_F_6_ molecules, thereby reinforcing C_3_F_6_ affinity.

A more sophisticated strategy involves tailoring the geometry of pore cages rather than simply controlling aperture size. Ma et al. constructed a cage-based MOF (JXNU-22) using [Fe_2_Co(μ_3_-O)] clusters bridged by tetrazolate and pyridyl linkers, featuring trigonal bipyramidal and cylindrical cages [127]. By replacing one linker with a methyl-substituted analog, they achieved an excellent size-exclusion effect for C_3_F_8_, producing C_3_F_8_ with >99.999% purity and a productivity of 321.7 L·kg^−1^ [127].

**Figure 9:**
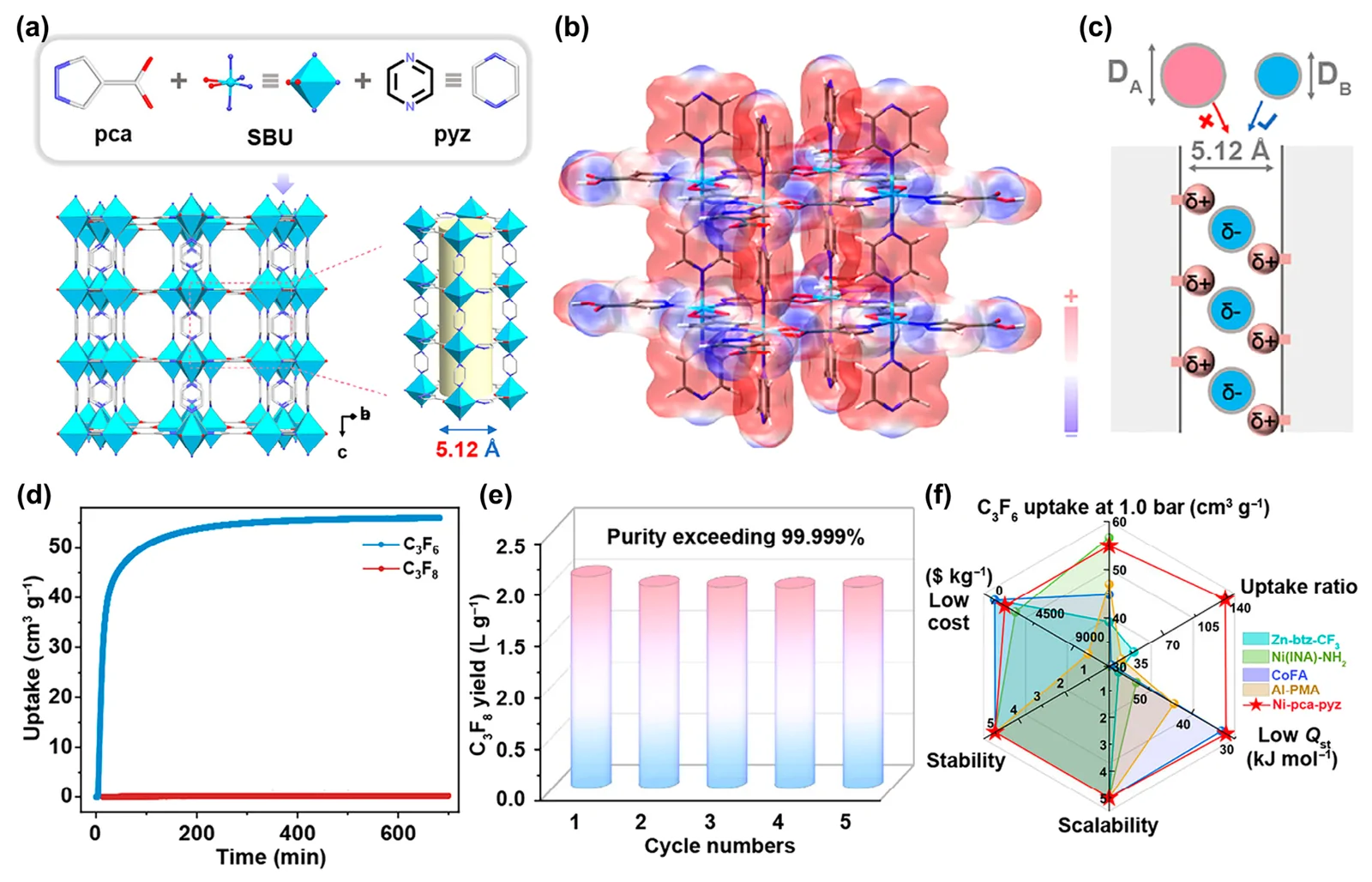
(**a**) Inorganic and organic building units and the crystal structure of Ni-pca-pyz (pca = 4-pyrazolecarboxylate anion; pyz = pyrazine). The secondary building unit (SBU) is highlighted as a blue hexahedral structure, serving as the node that connects into the overall MOF framework. The dashed line connects the MOF structure to the enlarged unit, in which the yellow cylinder represents the 1D hollow channel with a diameter of approximately 5.12 Å. (**b**) Electrostatic potential mapping of the channel interior of Ni-pca-pyz. The blue and pink regions denote negative and positive electrostatic potential, respectively, as indicated by the color scale bar. (**c**) Schematic representation of pore shape and electrostatic potential matching effect for C_3_F_8_ exclusion (pink ball: C_3_F_8_, the diameter is D_A_; blue ball: C_3_F_6_, the diameter is D_B_) (D_B_ < 5.12 Å < D_A_). (**d**) The kinetic adsorption curves of C_3_F_6_ (blue line) and C_3_F_8_ (red line) on Ni-pca-pyz at 298 K and 1.0 bar. (**e**) C_3_F_8_ productivity (purity exceeding 99.999%) over five consecutive breakthrough cycles. (**f**) Comparison of the comprehensive performance of the reported top-performing MOFs for C_3_F_6_/C_3_F_8_ separation. Adapted with permission from [130]. Copyright 2026 John Wiley & Sons, Inc.

Beyond pore geometry control, aligning the electrostatic potential of the pore surface with that of the target molecule offers an alternative pathway to achieving high selectivity. Xia et al. demonstrated this strategy in aluminum pyromellitate (Al-PMA) for C_3_F_6_/C_3_F_8_ separation [128]. The hydroxyl-lined channels (μ_2_-OH) create positive electrostatic traps that form strong hydrogen bonds with the smaller C_3_F_6_, while the aperture excludes the larger C_3_F_8_, yielding a C_3_F_8_ productivity of 173.8 cm^3^·g^−1^ with >99.999% purity [128]. Fang et al. extended this design to A520, a related Al-MOF with μ-OH-lined channels, achieving a record C_3_F_8_/N_2_ selectivity of 6034 [129]. Lan et al. further advanced this concept in Ni-pca-pyz [130]. The framework combines molecular-sieving channels that completely exclude C_3_F_8_ with electrostatic-potential complementary surfaces that enhance C_3_F_6_ recognition through multiple hydrogen-bonding interactions (Figure 9). This synergistic design yields a record C_3_F_6_/C_3_F_8_ uptake ratio of 137.6 and a C_3_F_8_ productivity of 2.06 × 10^3^ L·kg^−1^ with >99.999% purity [130]. Notably, these MOFs can be synthesized from commercially available reagents under environmentally friendly conditions, addressing scalability concerns for industrial adoption.

##### Hydrofluorocarbons (HFCs)

HFCs contain C–H bonds that impart weak polarity and hydrogen-bonding capability, providing additional interaction modes beyond those available to PFCs. Research on HFC adsorption can be categorized into two directions: (i) recovering individual constituents from azeotropic blends; (ii) developing adsorbents for adsorption-based cooling cycles [140].

The separation of azeotropic HFC blends, such as R-32(CH_2_F_2_)/R-125(C_2_HF_5_) (R-410A), exploits the subtle differences in both size- and polarity-based discrimination. Zeolites represent one class of materials that have proven effective for this task, as their performance is governed by cation composition and framework topology. Yancey et al. systematically investigated the adsorption of R-32 and R-125 on Linde Type A (LTA) zeolites with varying cation composition [131]. The substitution of Na^+^ with Ca^2+^ could enhance electrostatic interactions with the more polar R-32, increasing adsorption capacity and breaking the azeotropic equilibrium of R-32 and R-125 [131]. He also demonstrated that this cation-exchange strategy outperforms purely siliceous zeolites, where separation proceeds through molecular size discrimination within ~5.5 Å channels [141].

Pore volume is also an important structural parameter for HFC adsorption. Sosa et al. evaluated a series of commercial activated carbons and established that ultramicropore volume correlates most strongly with HFC uptake [142]. However, the limited chemical functionality of carbon surfaces restricts their ability to modulate binding strength. Mixed-ligand MOFs provide a complementary strategy that simultaneously tailors pore volume and pore chemistry. Through organic-linker modification and variation, a series of isostructural mixed-ligand MOFs (LIFM-66, 66/67-mix, and 67) were constructed [132], introducing different proportions of functional groups into the pore nanospaces with tunable surface areas. Adsorption measurements of hydrofluorocarbons (HFCs) and hydrochlorofluorocarbons (HCFCs) on these MOFs revealed a record-high uptake of R134a (a hydrofluorocarbon HFC, CH_2_FCF_3_, 1.09–1.14 g·g^−1^) and an ultrahigh uptake of R22 (a hydrochlorofluorocarbon HCFC, CHClF_2_, 0.85–0.96 g·g^−1^) under ambient conditions [132]. Notably, the adsorption performance for the greenhouse gases CO_2_, R134a, and R22 can be finely regulated by the introduction of methyl groups.

**Figure 10:**
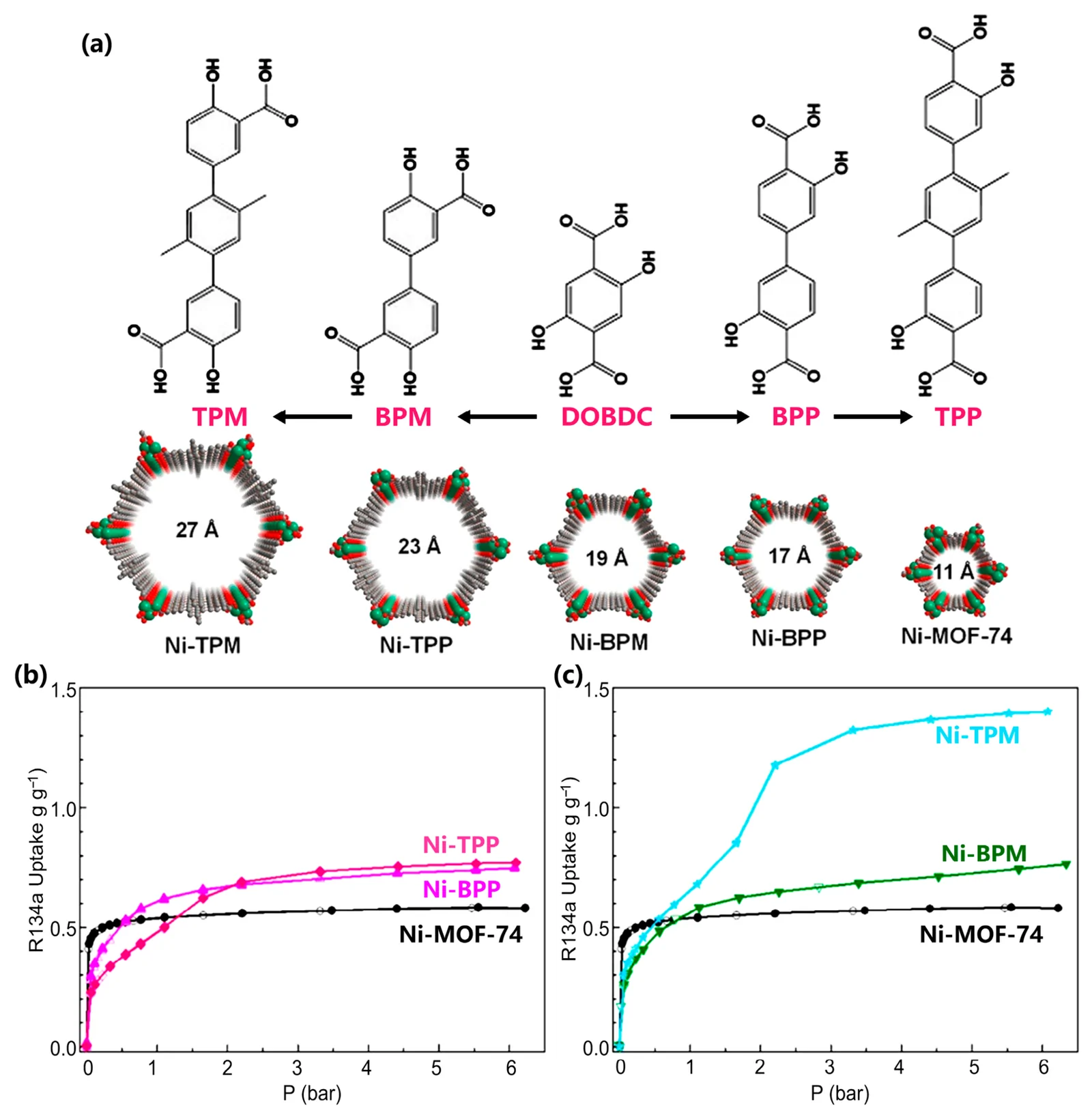
(**a**) Pore-engineering of basic Ni-MOF-74 by different ligands. Five isoreticular frameworks (Ni-MOF-74, Ni-BPP, Ni-BPM, Ni-TPP, and Ni-TPM) with pore apertures ranging from 11 Å to 27 Å were synthesized by varying the number and stereochemistry of phenylene rings in the organic linkers (TPP, BPP, DOBDC, BPM, TPM). (**b**,**c**) Equilibrium adsorption (solid) and desorption (open) R134a isotherms for different series of pore-engineered MOFs at 298 K: (**b**) para-series and (**c**) meta-series. In (**b**), the purple and red curves represent Ni-BPP and Ni-TPP, respectively. In (**c**), the green and blue curves represent Ni-BPM and Ni-TPM, respectively. The black curves in both panels denote Ni-MOF-74 as a reference. Adapted with permission from [133]. Copyright 2020 American Chemical Society.

For adsorption cooling applications, the design imperative balances three competing factors, which are high working capacity, facile regeneration, and rapid thermal cycle times. Zheng et al. systematically addressed this balance through ligand engineering in a Ni-MOF-74 series. They synthesized five isoreticular frameworks (Ni-MOF-74, Ni-BPP, Ni-BPM, Ni-TPP, and Ni-TPM) with pore apertures ranging from 11 Å to 27 Å by varying the number and stereochemistry of phenylene rings in the organic linker (Figure 10) [133]. Adsorption measurements for R134a revealed that the uptake increased progressively as the pore size increased. Ni-TPM (27 Å) exhibited the highest R134a uptake (1.12 g·g^−1^), a nearly twofold enhancement over Ni-MOF-74 with the smallest pore size. In situ infrared spectroscopy and DFT calculations further elucidated that the binding is based on the coordination of R134 with unsaturated Ni^2+^ sites through its fluorine atoms at low pressures. This work demonstrates that pore size and metal-site accessibility must be co-optimized to achieve both high working capacity and energy-efficient regeneration for MOF-based HFC cooling pairs.

Recently, HOFs have also emerged as a complementary platform for HFC-related separations. Ji et al. rationally synthesized a stable microporous HOF (HOF-TDBB), using a hexacarboxylic acid ligand (H_6_TDBB) as the building block [135]. The framework self-assembles through extensive hydrogen-bonding interactions between carboxyl groups, forming a rigid porous architecture with a unique dual-pore structure. The pore surface is decorated with abundant aromatic rings and high-density carboxylate oxygen atoms, creating an electrostatic-potential-matching environment that preferentially interacts with hydrofluoroethanes. In static adsorption experiments at 298 K and 1 bar, HOF-TDBB shows significantly higher uptakes for CF_3_CH_2_F (109.9 cm^3^·g^−1^) and CF_3_CHF_2_ (95.0 cm^3^·g^−1^) than for C_2_F_6_ (50.3 cm^3^·g^−1^). The breakthrough test yields 11.7 mol kg^−1^ of high-purity (>99.99%) C_2_F_6_ from a ternary CF_3_CH_2_F/CF_3_CHF_2_/C_2_F_6_ mixture, and the separation performance is maintained over dozens of consecutive cycles. Moreover, HOF-TDBB can be rapidly synthesized on a large scale via simple rotary evaporation and also has exceptional stability in air, common organic solvents, and even under harsh conditions like boiling water and strong acids. This work demonstrated that HOFs, traditionally limited by stability concerns, can overcome these challenges through rational design, offering a scalable and robust alternative to MOFs for industrial HFC purification.

##### Chlorofluorocarbons (CFCs) and Hydrofluoroolefins (HFOs)

CFCs and HFOs share a common design challenge: both possess asymmetric charge distributions that enable interactions with polar surfaces. CFCs possess strongly electronegative chlorine substituents, while HFOs possess electron-rich C=C double bonds.

The adsorption behavior of CFCs in zeolites has been examined through both theoretical and experimental approaches. Early computational work on CF_2_Cl_2_ in CsNaY zeolite revealed a two-stage uptake mechanism: preferential binding to Cs+ cations at low pressures, followed by denser molecular packing at elevated pressures [143]. A subsequent comparative study of NaY zeolite, MIL-127(Fe), and carbon nanotubes for CCl_2_F_2_/N_2_ separation established that selectivity originates primarily from enthalpy of adsorption disparity rather than steric constraints, with wider carbon nanotubes achieving selectivity on the order of 10^4^ [136]. Fluorinated Zr-MOFs have since provided experimental validation of fluorophilic pore environments, demonstrating high uptake capacities for various chlorocarbons and fluorocarbons through F–F and van der Waals interactions [144].

Experimental investigations of HFO adsorption remain less extensive than those for CFCs. The adsorption of HFO-1234yf on activated carbon Maxsorb III has been shown to reach 1.3 g·g^−1^ capacity with no hysteresis, confirming that physical pore-filling in high-surface-area carbons can achieve substantial HFO uptake without specific binding sites [137]. Computational studies have further revealed that HFCs exhibit stronger adsorption than HFOs in MOF-74, attributable to stronger interactions between open metal sites and polar C–H bonds relative to electron-rich C=C bonds. This difference suggests that temperature modulation could reverse the selectivity order in mixed-refrigerant streams [138].

Across the refrigerant family, the common design thread is precise pore engineering to match molecular dimensions, often combined with functional groups that exploit specific polar interactions. MOFs offer the greatest structural tailorability for sub-angstrom aperture control, zeolites provide robust electrostatic interactions through cation exchange, and activated carbons deliver cost-effective solutions where high surface area and ultramicropore volume suffice. The choice among these platforms depends on the specific separation target (azeotrope breaking, trace removal, or bulk recovery), and the associated trade-offs between capacity, selectivity, and regenerability.

#### 3.2.3. Other VFC Species

**Figure 11:**
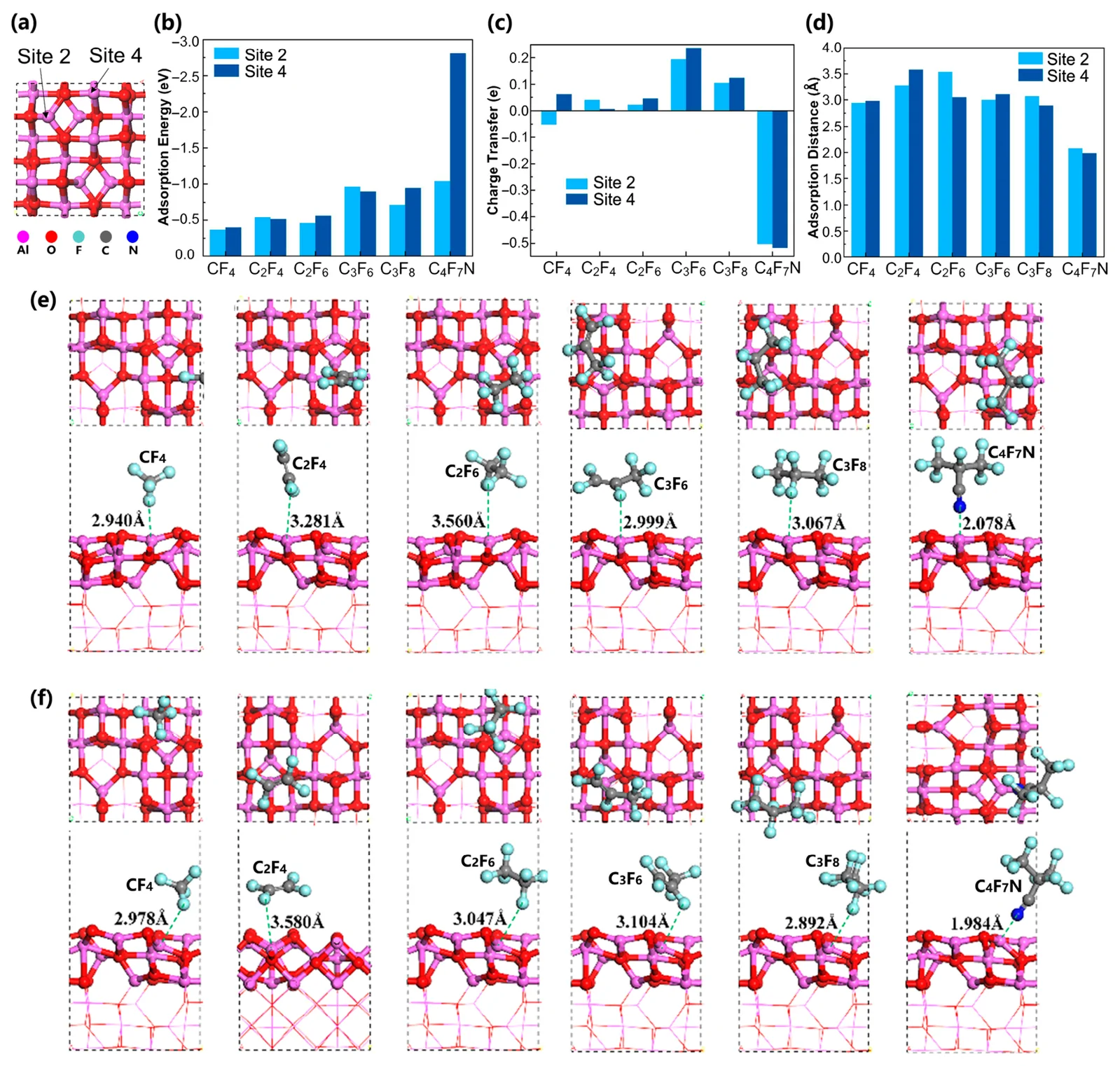
(**a**) Top view of the γ-Al_2_O_3_ surface. The calculated (**b**) Adsorption energy, (**c**) charge transfer energy, and (**d**) adsorption distance between the γ-Al_2_O_3_ surface and gases of CF_4_, C_2_F_4_, C_2_F_6_, C_3_F_6_, C_3_F_8_, and C_4_F_7_N. (**e**,**f**) Geometric structure of gases adsorbed on the γ-Al_2_O_3_ surface at (**e**) site 2 and (**f**) site 4. The adsorption configurations and the corresponding gas–surface distances (marked by dashed lines) differ significantly between site 2 and site 4. Adapted from Ref. [146].

Beyond the VFCs discussed above, a few other species have also attracted recent attention in the context of gas-insulated equipment. C_4_F_7_N has been treated as a promising eco-friendly alternative to SF_6_, yet under discharge or overheating faults, it decomposes into harmful by-products (CF_4_, C_2_F_6_, C_3_F_8_, and C_3_F_6_) that threaten equipment safety [145]. Xiao et al. systematically investigated the adsorption of C_4_F_7_N and its decomposition products on γ-Al_2_O_3_ using both experimental and DFT approaches (Figure 11) [146]. The study revealed that γ-Al_2_O_3_ exhibits stronger interactions with C_4_F_7_N, C_3_F_6_, and C_3_F_8_ than with CF_4_ and C_2_F_6_, with significant charge transfer confirming chemisorption. These findings suggest that γ-Al_2_O_3_ is viable for abatement of C_4_F_7_N in exhaust streams, though its pronounced desorption tendency renders it unsuitable for in situ capture of decomposition by-products within energized equipment.

Carbonyl fluoride (COF_2_) is a decomposition product of SF_6_ and C_4_F_7_N and also serves as a low-GWP cleaning gas candidate in semiconductor processes [147]. Unlike the inert perfluorinated VFCs discussed above, COF_2_ is highly reactive and readily hydrolyzes to form HF and CO_2_. This chemical reactivity leads to adsorption behavior that differs from conventional physical capture. Experimental studies on COF_2_ adsorption remain scarce. González Fá et al. employed DFT to evaluate pristine and boron-doped aluminum nitride nanosheets (AlNNSs) for COF_2_ adsorption [148]. The adsorption energy reached as low as −3.44 eV on the B-doped AlNNS. It is significantly stronger than typical physisorption (<−0.5 eV), indicating a strong chemical interaction. Pronounced changes in the energy gap and work function upon COF_2_ adsorption suggest that B-doped AlNNSs could serve as an effective adsorbent. While the computational results provide a compelling mechanistic framework, definitive experimental evidence awaits further investigation.

### 3.3. Comparative Assessment of Porous Adsorbents and Practical Considerations

The above sections have revealed that different porous platforms have worked for multiple VFC families. Table 3 provides a cross-cutting summary of representative sensing and capture mechanisms for each major VFC class, highlighting the shared design principles for functionalities. Metal oxides and conductive frameworks have been the primary focus, with detection capabilities demonstrated for SF_6_, NF_3_, and C_4_F_7_N. MOFs offer unparalleled structural precision for SF_6_ and NF_3_ capture, zeolites provide robust and cost-effective solutions for HFC separations, while carbon adsorbents excel in scalability and ultramicropore engineering for perfluorinated gases. COFs and the emerging HOFs and POCs represent complementary platforms that address specific selectivity challenges, though their practical deployment remains at an earlier stage. However, these promising approaches still do not translate into a clear hierarchy of material suitability for industrial VFC management.

For sensing applications, many reported detection limits remain orders of magnitude above the sub-ppm regulatory targets that define practical monitoring needs. This gap reflects that most studies to date have focused on demonstrating proofs of concept rather than evaluating performance under realistic operating conditions. Critical issues, such as humidity interference, long-term baseline drift, and selectivity against coexisting gases, remain largely underexplored.

For capture applications, irreversible adsorption, while enabling high uptake (such as Co-MOF-74 for NF_3_), prevents regeneration and limits practical applications, raising concerns over sorbent disposal and secondary waste. The majority of reported adsorption capacities and selectivities are derived from single-component isotherms and IAST calculations under dry, idealized conditions. Systematic data on competitive adsorption in humid, multi-component streams, which more accurately represent real-world off-gas or ambient air, remain scarce. While cost-effectiveness and scalability are often mentioned in the reports, independent assessments, such as ligand synthesis cost, space–time yield, and pelletization or membrane fabrication, are seldom addressed in laboratory-scale studies. These considerations suggest that laboratory-scale performance, while essential for benchmarking and mechanistic understanding, does not yet directly translate into engineering readiness for VFC management.

## 4. Perspectives on Integrated “Sense-and-Capture” System for VFC Management

The preceding sections have surveyed porous materials for VFC sensing and capture as two largely independent tracks, each with distinct performance metrics and design priorities. Despite sharing the same host–guest chemistry foundation, sensing and capture have diverged in their optimization targets, which is difficult to reconcile in a single material. This difficulty is amplified by the intrinsic properties of VFCs. Their chemical inertness and minimal polarizability severely constrain both signal generation and adsorption affinity.

A conceptual framework for integrated VFC management can be envisioned as a sequential workflow from detection (sensing) to decision (signal processing) and response (capture), with feedback loops that enable adaptive operation. Despite CO_2_ or other VOCs, where charge transfer or acid–base interactions provide ready handles for integrated sensing and capture [149,150,151], VFCs demand fundamentally different design strategies that remain largely unexplored.

Gutiérrez et al. demonstrated a plasmonic Mg/MgO nanoparticle platform that simultaneously degrades and detects SF_6_ by the localized surface plasmon resonance (LSPR) [152]. The intense near-field enhancement and hot-carrier generation drive the decomposition of SF_6_ into non-hazardous products (MgF_2_ under UV irradiation and MgSO_4_ under visible light). Meanwhile, the perturbation of the local dielectric environment induces measurable LSPR spectral shifts, enabling real-time detection. This system can also be regenerated in seconds by hydrogen plasma treatment and reused in multiple cycles.

**Figure 12:**
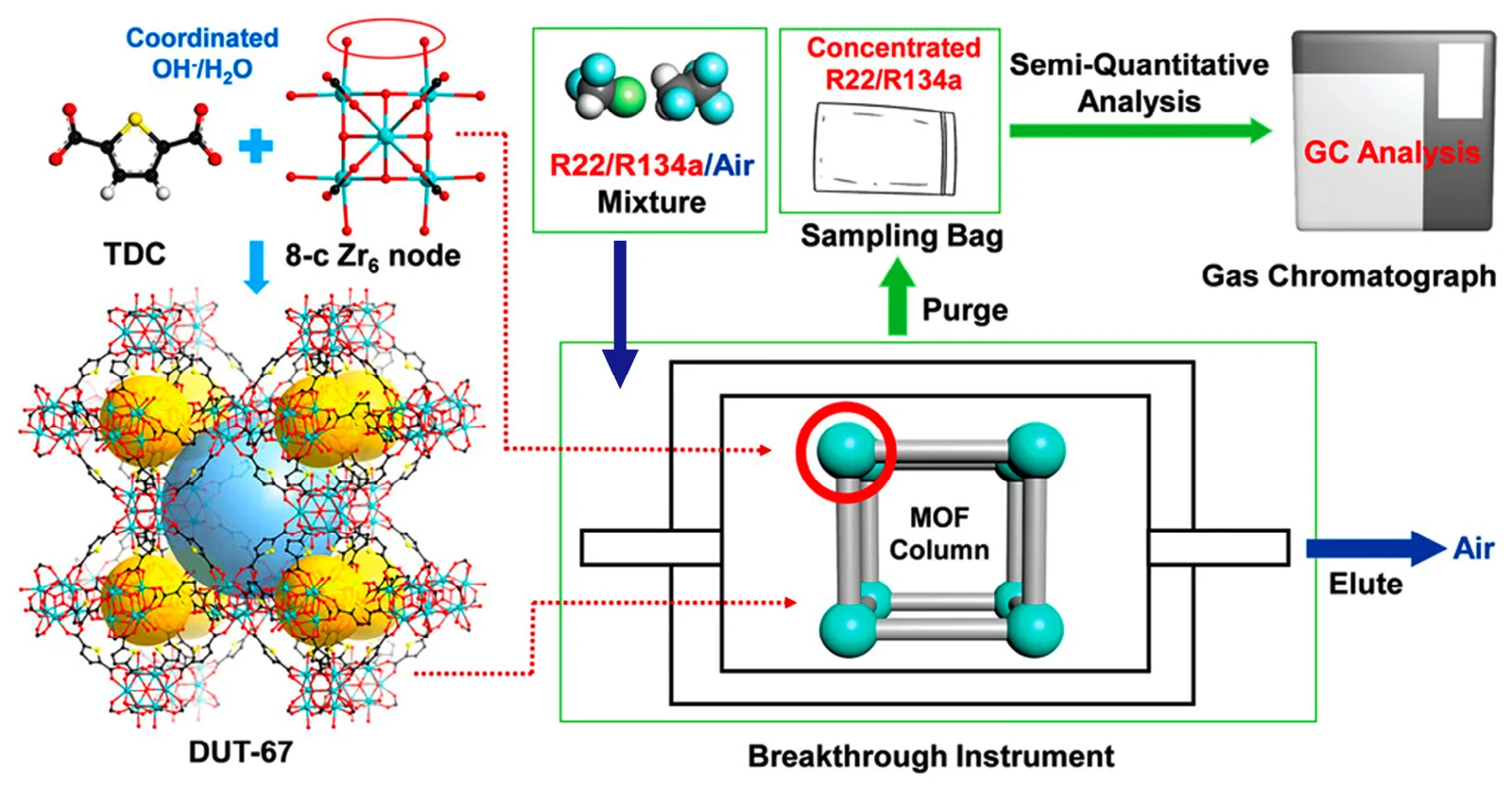
Diagram of a system integrating adsorption, separation, enrichment, and GC analysis for low concentrations of FC/CFCs using DUT-67 as a column adsorbent. The organic linker is TDC (2,5-thiophenedicarboxylic acid), in which the atoms are C (black), O (red), S (yellow) and H (white). In the 8-connected Zr_6_ node (8-c Zr_6_ node), the blue and red spheres denote Zr and O atoms, respectively. The square framework representing MOF structure is composed of green spheres (nodes) and gray rods (linker). The red circle highlights the 8-connected Zr_6_ node in the DUT-67 structure. The yellow and blue spheres in DUT-67 scheme emphasize the hollow cavities within the MOF framework. The red dashed arrows indicate the preparation process of the DUT-67 adsorbent; the solid arrows indicate the analytical/testing flow path of the integrated system. Adapted from Ref. [153].

Extending the concept of integration beyond a single pair of functions, Xiong et al. advanced a more holistic strategy by employing the robust Zr-based MOF, DUT-67 [153]. The framework of DUT-67 is characterized by a high density of coordinated water and hydroxyl groups bound to the Zr-clusters, in conjunction with a well-defined, ordered pore architecture. DUT-67 delivers high uptake capacities for both R22 (124 cm^3^·g^−1^) and R134a (116 cm^3^·g^−1^), along with exceptional selectivity over CO_2_ that persists under both low-concentration and humid conditions. Taking advantage of the high adsorption selectivity, rapid kinetics, moisture tolerance, facile regeneration, and recyclability of DUT-67, a semi-quantitative analytical protocol was further established. These findings illustrate that a single MOF platform can transcend mere capture, enabling a comprehensive “capture–separation–analysis” workflow for chlorofluorocarbons under ambient conditions (Figure 12).

Looking forward, the realization of integrated sense-and-capture systems for VFCs hinges on reconciling the opposing thermodynamic requirements of capture (strong binding) and sensing (reversible, signal-generating interactions), a challenge that spans three interdependent levels of design.

At the molecular level, a central question is whether host–guest interactions can be engineered to simultaneously meet the conflicting demands of capture and sensing. Physisorption (with binding energies typically in the range of 5–40 kJ mol^−1^) can afford the reversibility needed for sensing, whereas effective capture generally necessitates stronger interactions that operate beyond this regime. A systematic approach would involve varying the density and arrangement of polarizing groups within a series of isoreticular frameworks and correlating the binding energies with signal–response kinetics. Frameworks with flexible pores or stimuli-responsive linkers offer a platform to test whether analyte-induced conformational changes can produce spectroscopic readouts decoupled from charge-transfer pathways, circumventing the intrinsically weak electronic coupling of most VFCs. Dynamic covalent chemistry may offer a complementary route. Reversible covalent bonds can undergo exchange or rearrangement in response to guest binding, potentially bridging the gap between strong adsorption and reversible signal transduction. This approach may transcend the conventional distinction between physisorption and chemisorption, offering new handles for designing VFC-responsive materials that are both sensitive and regenerable.

At the transducer level, the conflicting requirements of sensing sensitivity (favored by thin, diffusion-accessible films) and capture capacity (favored by high sorbent mass) suggest that monolithic architecture may be inherently limiting. Possible resolutions include hierarchical or gradient structures that spatially partition sensing and capture functions, or transduction mechanisms that probe binding events through orthogonal physical channels (e.g., mass-loading vs. impedance changes), extracting both concentration and binding information from the same material. Emerging platforms such as HOFs and POCs may offer unique advantages in this context. For instance, the solution processability of HOFs could facilitate the fabrication of uniform thin films for integrated devices.

At the system level, closed-loop operation may be achieved through coordination of distinct, independently optimized sensing and capture modules. In such a configuration, a sensitive detection module continuously monitors VFC concentration and triggers a high-capacity capture module only upon exceeding a threshold. This decouples the performance requirements of the two functions, allowing each to be optimized without compromise, while remaining compatible with existing industrial safety infrastructure. VFC management can thus evolve from periodic monitoring to condition-based intervention, reducing the latency between leak onset and response.

## 5. Conclusions and Outlook

This review provides a systematic account of porous materials for the detection and capture of VFCs. We have shown that rational design of pore architecture and surface chemistry provides a versatile means to overcome the intrinsic inertness of these compounds. In sensing, the emphasis lies on transducing weak host–guest interactions into measurable electrical, optical, or mechanical signals. In capture, the focus shifts to maximizing capacity, selectivity, and regenerability. Although both fields share a common foundation, their performance metrics and optimization targets have diverged considerably.

The very stability and inertness that make most VFCs valuable also hinder their detection and capture. These properties limit both adsorption strength and signal response, making conventional design strategies less effective. However, recent advances in framework chemistry have demonstrated that precise pore engineering and surface functionalization can overcome these limitations. Recognition environments can be tailored to discriminate individual VFC species, from polar gases to refrigerants with subtle molecular differences.

Despite these advances, the translation of laboratory-scale performance into practical VFC management remains limited. Most reported data are obtained under idealized conditions, while scalability, long-term stability, and real-world sensing requirements remain insufficiently addressed. The integration of sensing and capture within a unified material platform also faces challenges. The fundamental difficulty lies in reconciling the opposing thermodynamic requirements of the two functions for chemically inert analytes. Recent proof-of-concept systems have begun to explore this convergence, but the signal–capacity trade-off remains largely unresolved. Progress toward integrated systems will require advances across three interdependent levels: engineering host–guest interactions at the molecular level, designing device architectures at the transducer level, and coordinating intelligent modules at the system level. These directions are not sequential but coupled, and their pursuit promises to transform VFC management from a reactive, monitoring-based practice into a proactive, condition-driven safety paradigm.

## Figures and Tables

**Table 1:** Representative porous materials for SF_6_ and NF_3_ capture: structural features and adsorption performance.

Adsorbent	Key Structural Features	SF_6_ Uptake/ Selectivity	NF_3_ Uptake/ Selectivity	Adsorption Data Type	Ref.
MOFs					
DMOF-4Cl	Pore size 6.5 Å; BET surface area 1023 m^2^·g^−1^; chlorination modification	2.0 mmol·g^−1^; SF_6_/N_2_ (1/9, *v*/*v*) selectivity 122 (298 K, 1 bar)	-	Cal. (IAST); Exp. (sorption isotherms, breakthrough)	[96]
Ni(AIN)_2_	–NH_2_ networks with moisture stability; pore size 4.6 × 4.6 Å; BET surface area 674 m^2^·g^−1^	-	~55 cm^3^·g^−1^; NF_3_/N_2_ (1/9, *v*/*v*) selectivity ~25; (298 K,101 kPa)	Cal. (IAST); Exp. (sorption isotherms, breakthrough)	[97]
UiO-67(Zr)	Dual pore size 12 and 23 Å; BET surface area 2411 m^2^·g^−1^	9.66 mmol·g^−1^ (298 K, 1 bar); SF_6_/N_2_ (1/9, *v*/*v*) selectivity 37 (298 K,10 bar)	-	Cal. (IAST); Exp. (sorption isotherms)	[98]
Co-MOF-74	Pore size 11 Å; BET surface area 1313.4 m^2^·g^−1^; open metal sites	-	54.0 cm^3^·g^−1^ (chemisorption); NF_3_/N_2_ (1/9, *v*/*v*) selectivity 299.6 (298 K, 1 bar)	Cal. (IAST); Exp. (sorption isotherms)	[99]
CALF-20	Pore size 4.8 Å; BET surface area 458 m^2^·g^−1^; cyclability (stable 5 cycles)	-	51.1 cm^3^·g^−1^ (273 K, 1 bar); NF_4_/CF_4_ (1/1, *v*/*v*) selectivity ~4 (298 K, 1 bar)	Cal. (IAST); Exp. (sorption isotherms, breakthrough)	[100]
ATC-Cu	Rectangular channels (4.43 × 5.39 Å^2^); BET surface area 703 m^2^·g^−1^; overlapping electric fields		63.9 cm^3^·g^−1^; NF_3_/CF_4_ (1/1, *v*/*v*) selectivity 2.16 (298 K, 1 bar)	Cal. (IAST); Exp. (sorption isotherms, breakthrough)	[101]
Al-PMOF	A 3D porous network (elliptical pores 6 × 11 Å and rectangular pores 5 Å); BET surface area 1271 m^2^·g^−1^; abundant Al–O cluster polar sites	6.15 mmol·g^−1^; SF_6_/N_2_ (1/9, *v*/*v*) selectivity 581 (298 K, 1 bar)	3.00 mmol·g^−1^; NF_3_/N_2_ (1/9, *v*/*v*) selectivity 18.2 (298 K, 1 bar)	Cal. (IAST); Exp. (sorption isotherms, breakthrough)	[102]
Zeolite					
K-Y zeolite	FAU framework; pore size 7.4 Å; BET surface area 458 m^2^·g^−1^	1.94 mmol·g^−1^; dynamic SF_6_/N_2_ (1/9, *v*/*v*) selectivity 97.0 (298 K, 0.1 bar)	-	Exp. (breakthrough)	[103]
W-MFI	Pore size 5.7 Å; BET surface area 451.7 m^2^·g^−1^; Lewis acid sites	44.6 mL·g^−1^; SF_6_/N_2_ (1/99, *v*/*v*) selectivity 260 (298 K,1 bar)	30.8 mL·g^−1^; NF_3_/N_2_ (1/99, *v*/*v*) selectivity 25.3 (298 K,1 bar)	Cal. (IAST); Exp. (sorption isotherms, breakthrough)	[104]
Porous Carbons					
LG-750-3	Lignin-derived; pore size 5.8 Å; BET surface area 1609 m^2^·g^−1^; cyclability (stable 5 cycles)	2.66 mmol·g^−1^; SF_6_/N_2_ (1/9, *v*/*v*) selectivity 635 (298 K,1 bar)	-	Cal. (IAST); Exp. (sorption isotherms, breakthrough)	[105]
PC-750	Dense micropore (7 Å) with mesopore; BET surface area 1392.8 m^2^·g^−1^	4.09 mmol·g^−1^; SF_6_/N_2_ (1/9, *v*/*v*) selectivity 436.4 (298 K, 1 bar)	2.27 mmol·g^−1^ NF_3_/N_2_ (1/9, *v*/*v*) selectivity 61.4 (298 K, 1 bar)	Cal. (IAST); Exp. (sorption isotherms, breakthrough)	[106]
ACK1	KOH-modified; pore size 5–9 Å; 97.6% microporosity; BET surface area 1053 m^2^·g^−1^	3.10 mmol·g^−1^; SF_6_/N_2_ (1/9, *v*/*v*) selectivity 683.9 (298 K,1 bar)	2.09 mmol·g^−1^ NF_3_/N_2_ (1/9, *v*/*v*) selectivity 29.5 (298 K,1 bar)	Cal. (IAST); Exp. (sorption isotherms, breakthrough)	[107]
ACK1-800	K_2_CO_3_-activated; pore size 7 Å; BET surface area 1167 m^2^·g^−1^; humidity tolerance	4.35 mmol·g^−1^ (298 K,1 bar)	-	Exp. (sorption isotherms, breakthrough)	[108]
PVDF-800	Pore size 6.8 Å; BET surface area 1089 m^2^·g^−1^	1.93 mmol·g^−1^ (298 K, 0.1 bar); SF_6_/N_2_ (1/9, *v*/*v*) selectivity about 350 (298 K,1 bar)	0.57 mmol·g^−1^ (298 K, 0.1 bar); NF_3_/N_2_ (1/9, *v*/*v*) selectivity about 28 (298 K,1 bar)	Cal. (IAST); Exp. (sorption isotherms, breakthrough)	[109]
AC-KOH(1:1)-800	KOH-activated chitosan; 0.5–1.0 nm micropores; BET surface area 1852 m^2^·g^−1^; cyclability (stable 8 cycles)	5.88 mmol·g^−1^; SF_6_/N_2_ selectivity 126 (298 K, 1 bar)	-	Cal. (IAST); Exp. (sorption isotherms, breakthrough)	[110]
KHP-800	Pore size 5.2–8.0 Å; BET surface area 1672 m^2^·g^−1^; cyclability (stable 5 cycles)	2.41 mmol·g^−1^; SF_6_/N_2_ uptake ratio 5.2 (298 K, 0.1 bar)	-	Exp. (sorption isotherms, breakthrough)	[111]
COFs					
COF-2O	Heteroatom functionalization; pore size 8.5 Å; ASA 2158.62 m^2^·g^−1^	6.44 mmol·g^−1^; SF_6_/N_2_ (1/9, *v*/*v*) selectivity 400.79 (298 K, 1 bar)	-	Sim. (GCMC)	[112]
RCOF-1	Pore size 9.0 Å; BET surface area 1139 m^2^·g^−1^; cyclability (stable 5 cycles)	3.46 mmol·g^−1^; SF_6_/N_2_ (1/9, *v*/*v*) selectivity 83 (298K, 1 bar)	-	Cal. (IAST); Exp. (sorption isotherms, breakthrough)	[113]
CPOF-12	A 3D COF; *tert*-butyl functionalization; pore size 5.9 Å; BET surface area 1140 m^2^·g^−1^; cyclability (stable 5 cycles)	2.20 mmol·g^−1^; SF_6_/N_2_ (1/9, *v*/*v*) selectivity 149.4 (298 K, 1 bar)	-	Cal. (IAST); Exp. (sorption isotherms, breakthrough)	[114]
POCs					
CC3α	Flexible molecular crystal; window diameter ~3.6 Å	Three SF_6_ molecules per cage; SF_6_/N_2_ (1/9, *v*/*v*) selectivity 76.5 (298 K, 1 bar)	-	Cal. (IAST) Exp. (sorption isotherms, breakthrough)	[115]
TC1	Triangular windows ~1.4 nm; BET surface area 1157 m^2^·g^−1^; multiple triazine and tetrazine moieties; cyclability (stable 5 cycles)	2.59 mmol·g^−1^; SF_6_/N_2_ (1/9, *v*/*v*) selectivity 135 (298 K, 1 bar)	-	Cal. (IAST); Exp. (sorption isotherms, breakthrough)	[116]

**Table 2:** Representative porous materials for refrigerant capture, organized by gas family, porous structural features, and adsorption performance.

Gas Family	Adsorbent	Key Structural Features	Adsorption Performance	Adsorption Data Type	Ref.
PFCs	Ni(ADC)(DABCO)_0.5_	Anthracene-functionalized; pore size 5.2 Å; BET surface area 712 m^2^·g^−1^; cyclability (stable 5 cycles)	CF_4_ uptake 0.52 mmol·g^−1^ (298 K, 0.1 bar); CF_4_/N_2_ (1/9, *v*/*v*) selectivity 23 (298 K, 1 bar)	Cal. (IAST); Exp. (sorption isotherm, breakthrough)	[120]
	Zn(fba)	Feature 1D channels with size 6.0 Å; BET surface area 345 m^2^·g^−1^; water stable	CF_4_ uptake 1.05 mmol g^−1^; CF_4_/N_2_ (1/9, *v*/*v*) selectivity 29 (298 K, 1 bar)	Cal. (IAST); Exp. (sorption isotherm, breakthrough)	[121]
	PFC-cage (F-cage)	Perfluorinated side chains; cage size ~1.3 nm; BET surface area 752 m^2^·g^−1^; cyclability (stable 7 cycles)	c-C_4_F_8_ uptake 1.66 mmol g^−1^; c-C_4_F_8_/N_2_ (1/99, *v*/*v*) selectivity 4385 (313 K, 1 bar);	Cal. (IAST); Exp. (sorption isotherm, breakthrough)	[122]
	Al-Fum	Pore size 6.8 Å; BET surface area 1085 m^2^·g^−1^; cyclability (stable 5 cycles)	C_2_F_6_ uptake 3.30 mmol·g^−1^; C_2_F_6_/N_2_ (1/9, *v*/*v*) selectivity 299.6 (298 K, 1 bar)	Cal. (IAST); Exp. (sorption isotherm, breakthrough)	[123]
	Zn(BPZ)	Open metal sites; pore size 5.6 × 5.6 Å^2^; BET surface area 862 m^2^·g^−1^; cyclability (stable 5 cycles)	C_2_F_6_ uptake 2.90 mmol·g^−1^; C_2_F_6_/N_2_ (3/97, *v*/*v*) selectivity 24.8 (298 K, 1 bar)	Cal. (IAST); Exp. (sorption isotherm, breakthrough)	[124]
	Zn-bzc-CF_3_	–CF_3_ functionalized; window aperture size 5.13 × 4.84 Å^2^; cyclability (stable 5 cycles)	C_2_F_6_ uptake 47 cm^3^·g^−1^ (298 K, 1 bar)	Exp. (sorption isotherm, breakthrough)	[125]
	Ni(INA)_2_-NH_2_	–NH_2_ functionalized channels; window aperture size 5.63 × 5.12 Å^2^; BET surface area 436.9 m^2^·g^−1^; cyclability (stable 5 cycles)	C_3_F_6_ uptake 56.7 cm^3^·g^−1^; C_3_F_8_ uptake 5.5 cm^3^·g^−1^ (298 K, 1 bar)	Exp. (sorption isotherm, breakthrough)	[126]
	JXNU-22(Me)	Cage-based MOF; methyl-substituents; cages size 5.4–7.8 Å; BET surface area 1828.2 m^2^·g^−1^; cyclability (stable 5 cycles)	C_3_F_6_ uptake 138.3 cm^3^·g^−1^; C_3_F_6_/C_3_F_8_ (1/9, *v*/*v*) selectivity about 9.2 (298 K, 1 bar)	Cal. (IAST); Exp. (sorption isotherm, breakthrough)	[127]
	Al-PMA	Hydroxyl-lined channels (μ_2_-OH); aperture size 5.3 × 4.7 Å^2^; BET surface area 394 m^2^·g^−1^; cyclability (stable 5 cycles)	C_3_F_6_ uptake 39.2 cm^3^·g^−1^; C_3_F_8_ uptake 1.06 cm^3^·g^−1^ (298 K, 1 bar)	Exp. (sorption isotherm, breakthrough)	[128]
	A520 (Al-MOF)	Feature 1D channels lined with μ-OH and fumarate; aperture size 5.7 × 6.0 Å^2^; BET surface area 1049 m^2^·g^−1^; cyclability (stable 5 cycles)	C_3_F_8_ uptake 65.6 cm^3^·g^−1^; C_3_F_8_/N_2_(1/9, *v*/*v*) selectivity 6034 (298 K, 1 bar)	Cal. (IAST); Exp. (sorption isotherm, breakthrough)	[129]
	Ni-pca-pyz	Pore size 5.12 Å; BET surface area 1015.91 m^2^·g^−1^; cyclability (stable 5 cycles)	C_3_F_8_ uptake 55.02 cm^3^·g^−1^; C_3_F_6_/C_3_F_8_ uptake ratio 137.6 (298 K, 1 bar)	Exp. (sorption isotherm, breakthrough)	[130]
HFCs	Zeolite 5A (Ca^2+^-exchanged)	LTA framework (5 Å); open metal sites (Ca)	HFC-32 uptake 4.99 mmol·g^−1^ (298 K, 1 bar); HFC-32/HFC-125 selectivity 9.6–10.9 (25–75 mol% HFC-125)	Cal. (IAST); Exp. (sorption isotherm)	[131]
	LIFM-66	Pore size 1.62 nm; BET surface area 3631 m^2^·g^−1^	R134a uptake 1.09–1.14 g·g^−1^; R134a/N_2_ selectivity (1/99, *v*/*v*) (298 K, 1 bar)	Cal. (IAST); Exp. (sorption isotherm, breakthrough)	[132]
	Ni-TPM	Open metal sites (Ni); pore size 27 Å; BET surface area 2420 m^2^·g^−1^	HFC-134a uptake 1.12 g·g^−1^ (298 K, 1 bar)	Exp. (sorption isotherm)	[133]
	Phenol resin-derived carbon	Average pore diameter 1.62 nm; BET surface area 2992 m^2^·g^−1^	HFC-32 uptake 2.34 g·g^−1^ (303 K, 1 bar); no hysteresis	Exp. (sorption isotherm)	[134]
	HOF-TDBB	Abundant aromatic rings and carboxylate oxygen atoms at pore surface; pore size 9.6–9.8 Å; BET surface area 1346.2 m^2^·g^−1^; cyclability (stable 30 cycles)	CF_3_CH_2_F uptake 109.9 cm^3^·g^−1^; CF_3_CH_2_F/C_2_F_6_ selectivity 18 (298 K, 1 bar)	Cal. (IAST); Exp. (sorption isotherm, breakthrough)	[135]
CFCs/HFOs	CNT(11,11)	Armchair conformation; internal diameter 1.49 nm; metallic conductivity	CCl_2_F_2_/N_2_ selectivity up to 10^4^ (CNT(11,11), simulated)	Sim. (GCMC)	[136]
	Maxsorb III (activated carbon)	Pore size 2.0–2.14 nm; BET surface area ~3000 m^2^·g^−1^	HFO-1234yf uptake 1.3 g·g^−1^ (313 K, 300 kPa)	Exp. (sorption isotherm)	[137]
	Mg-MOF-74	Open metal site; channel size about 11 Å	R134a uptake about 0.70 g·g^−1^; R32 uptake about 0.65 g·g^−1^; R1234ze uptake about 0.61 g·g^−1^ (313 K, 10 bar)	Sim. (GCMC)	[138]

**Table 3:** Overview of representative sensing materials, mechanisms, and capture materials for each VFC class.

Gas Family	Representative Sensing Materials and Mechanisms	Representative Capture Materials	Shared Design Principles
SF_6_/NF_3_	SnO_2_, Co_3_O_4_, SnO_2_/MXene (chemiresistive)	MOFs, zeolites, carbons, COFs, POCs	Lewis acid sites, polarizability-based recognition, pore confinement
PFC	SnO_2_ (chemiresistive)	MOFs, POCs	Size-sieving, fluorophilic surfaces, molecular topology
HFC	MOF (QCM)	Zeolites, carbons, MOFs, HOFs	Polarity-based recognition, hydrogen bonding, pore engineering
CFC/HFO	-	Zeolites, fluorinated MOFs, carbons	Enthalpy-driven selectivity, fluorophilic interactions

## Data Availability

No new data were created or analyzed in this study. Data sharing is not applicable to this article.

## Abbreviations

The following abbreviations are used in this manuscript:

ADC	Anthracene-9,10-dicarboxylic acid
Al-Fum	Aluminum fumarate
AlNNS	Aluminum nitride nanosheet
ASA	Active surface area
ATC-Cu	Adamantane tetracarboxylate-copper
BPM	4,4′-dihydroxybiphenyl-3,3′-dicarboxylic acid
BPP	2,2′-dihydroxybiphenyl-5,5′-dicarboxylic acid
BPZ	4,4′-bipyrazole
bzc	5-(trifluoromethyl)-1H-pyrazole-4-carboxylic acid
CALF	Calgary framework
CC3	Covalent cage-3
CFC	Chlorofluorocarbon
COF	Covalent organic framework
CPOF	Covalent porous organic framework
Cu/AC	Copper-supported activated carbon
DABCO	1,4-diazabicyclo[2.2.2]octane
DFT	Density functional theory
DUT	Dresden University of Technology
Eu-FDA	Europium-furandicarboxylate
fba	4,4′-(hexafluoroisopropylidene)bis-benzoate
GCMC	Grand Canonical Monte Carlo
GWP	Global warming potential
H_4_ATC	1,3,5,7-adamantanetetracarboxylic acid
H_6_TDBB	1,3,5-tris (3′,5′-dicarboxy-[1,1′-biphenyl]-4-yl) benzene
hBN-graphene	Hexagonal boron nitride–graphene
HCFC	Hydrochlorofluorocarbon
HF	Hydrogen fluoride
HFC	Hydrofluorocarbon
HFC-32	Difluoromethane (R-32)
HFC-125	Pentafluoroethane (R-125)
HFC-134a	1,1,1,2-tetrafluoroethane (R-134a)
HFO-1234yf	2,3,3,3-tetrafluoroprop-1-ene (R-1234yf)
H_2_FDA (FDCA)	2,5-furandicarboxylic acid
HFO	Hydrofluoroolefin
HOF	Hydrogen-bonded organic framework
IAST	Ideal adsorbed solution theory
INA	Isonicotinic acid
KSP	Potassium hydrogen phthalate
LSPR	Localized surface plasmon resonance
LTA	Linde Type A
MIL	Matériaux Institut Lavoisier
MOF	Metal–organic frameworks
pca	Pyrazinecarboxylic acid
pyz	Pyrazine
PAS	Photoacoustic spectroscopy
PET	Polyethylene terephthalate
PFAS	Per- and polyfluoroalkyl substances
PFC	Perfluorocarbon
PFOA	Perfluorooctanoic acid
PMA	Pyromellitic acid
POC	Porous organic cage
PVDF	Polyvinylidene fluoride
QCM	Quartz crystal microbalance
RCOF	Reconstructed covalent organic framework
SBU	Secondary building unit
SNAr	Nucleophilic Aromatic Substitution
TAPM	Tetra(4-aminophenyl)methane
*t*Bu-TFPPy	7-*tert*-butyl-1,3,5,9-tetrakis(4-formylphenyl)pyrene
TPM	3,3″-dihydroxy-2′,5′-dimethyl-[1,1′:4′,1″-terphenyl]-4,4″-dicarboxylic acid
TPP	4,4″-dihydroxy-2′,5′-dimethyl-[1,1′:4′,1″-terphenyl]-3,3″-dicarboxylic acid
UV-vis	Ultraviolet–visible
VFC	Volatile fluorinated compound
VOC	Volatile organic compound
